# Mixed Methods EvAluation of the high-volume low-complexity Surgical hUb pRogrammE (MEASURE): a mixed methods study protocol

**DOI:** 10.1136/bmjopen-2024-086338

**Published:** 2024-04-19

**Authors:** Arabella Scantlebury, Peter Sivey, Zecharias Anteneh, Ben Ayres, Karen Bloor, Adriana Castelli, Ana Cristina Castro-Avila, Firoza Davies, Simon Davies, Karen Glerum-Brooks, Nils Gutacker, Pete Lampard, Amar Rangan, Ahmed Saad, Andrew Street, Jinglin Wen, Joy Adamson

**Affiliations:** 1 York Trials Unit, Department of Health Sciences, University of York, York, UK; 2 Centre for Health Economics, University of York, York, Yorkshire, UK; 3 St George's University Hospitals NHS Foundation Trust, London, UK; 4 Department of Health Sciences, University of York, York, Yorkshire, UK; 5 Centre for Health and Population Sciences, Hull York Medical School, Hull, UK; 6 Trauma and Orthopaedics, James Cook University Hospital, Middlesbrough, UK; 7 Opthamology, James Cook University Hospital, Middlesbrough, UK; 8 Hull York Medical School, Hull, UK; 9 LSE, London, UK

**Keywords:** HEALTH ECONOMICS, Health policy, Protocols & guidelines, ORTHOPAEDIC & TRAUMA SURGERY, QUALITATIVE RESEARCH, SURGERY

## Abstract

**Introduction:**

The waiting list for elective surgery in England recently reached over 7.8 million people and waiting time targets have been missed since 2010. The high-volume low complexity (HVLC) surgical hubs programme aims to tackle the backlog of patients awaiting elective surgery treatment in England. This study will evaluate the impact of HVLC surgical hubs on productivity, patient care and the workforce.

**Methods and analysis:**

This 4-year project consists of six interlinked work packages (WPs) and is informed by the Consolidated Framework for Implementation Research. *WP1*: Mapping current and future HVLC provision in England through document analysis, quantitative data sets (eg, Hospital Episodes Statistics) and interviews with national service leaders. *WP2*: Exploring the effects of HVLC hubs on key performance outcomes, primarily the volume of low-complexity patients treated, using quasi-experimental methods. *WP3*: Exploring the impact and implementation of HVLC hubs on patients, health professionals and the local NHS through approximately nine longitudinal, multimethod qualitative case studies. *WP4*: Assessing the productivity of HVLC surgical hubs using the Centre for Health Economics NHS productivity measure and Lord Carter’s operational productivity measure. *WP5*: Conducting a mixed-methods appraisal will assess the influence of HVLC surgical hubs on the workforce using: qualitative data (WP3) and quantitative data (eg, National Health Service (NHS) England’s workforce statistics and intelligence from WP2). *WP6*: Analysing the costs and consequences of HVLC surgical hubs will assess their achievements in relation to their resource use to establish value for money. A patient and public involvement group will contribute to the study design and materials.

**Ethics and dissemination:**

The study has been approved by the East Midlands—Nottingham Research Ethics Committee 23/EM/0231. Participants will provide informed consent for qualitative study components. Dissemination plans include multiple academic and non-academic outputs (eg, Peer-reviewed journals, conferences, social media) and a continuous, feedback-loop of findings to key stakeholders (eg, NHS England) to influence policy development.

**Trial registration:**

Research registry: Researchregistry9364 (https://www.researchregistry.com/browse-the-registry%23home/registrationdetails/64cb6c795cbef8002a46f115/).

**Strengths and limitations of this study**

- The largest, independent, longitudinal, mixed-methods study of the high-volume low complexity (HVLC) surgical hub programme in England.
- Aims to identify the impact of HVLC surgical hubs on outcomes, productivity, patient care and the workforce and the differential impact of different service models of HVLC surgical hubs.
- Our flexible study design and application of implementation theory will ensure our findings capture the local context and are relevant to current policy.
- Quantitative analysis will be constrained by the quantity and quality of available information about the implementation and management of HVLC surgical hubs.
- Models of HVLC surgical hubs are expected to be locally tailored and the policy landscape is complex and shifting; this may present a challenge for analysis.

## Introduction

Globally, health services are experiencing unprecedented levels of demand and are struggling to recover from the effects of the COVID-19 pandemic.^1–4^ During 2020, approximately 34% of elective care was cancelled in the UK with elective hip replacements falling to almost zero between March and April 2020.^1 5^ The pandemic came against a backdrop of worsening performance against England’s 18-week target for elective surgery since 2011,^6^ and improvement post-pandemic has been variable.

National Health Service (NHS) England has launched an elective recovery plan^2^ designed specifically to address the ‘backlog’ of patients awaiting routine surgical treatment and to increase elective throughput of the NHS to 130% of pre-pandemic ‘business as usual’ volume by 2024/2025.^2^ The high-volume, low-complexity (HVLC) surgical hubs model is part of NHS England’s elective recovery blueprint, and has been specifically designed to reduce waiting lists for elective surgery in England. By ring-fencing staff and facilities in designated locations, the HVLC surgical hub programme aims to improve efficiency and productivity in the elective pathway by focusing efforts exclusively on treating low complexity elective patients more quickly. This contrasts with the usual model of elective care where patients with less complex conditions are treated in acute hospitals where surgeons, anaesthetists, nursing and diagnostic staff have competing demands from more complex cases and patients requiring urgent and emergency care.^7^

As of February 2024, NHS England reported that there were over 90 HVLC surgical hubs nationally. This includes ‘long-standing’ HVLC hubs that have been operational since before the official launch of the programme in 2022 and ‘new’ surgical hubs established subsequently. In 2023, the Royal College of Surgeons in collaboration with the Royal College of Anaesthetists developed a non-mandatory accreditation scheme, which aims to ensure national standards for HVLC surgical hubs, with 31 hubs receiving accreditation as of January 2024. Plans for the HVLC programme continue to evolve. It is expected that in addition to further HVLC surgical hubs being set-up and accredited that the type of patients directed to HVLC surgical hubs for treatment will expand. This evolution is designed to ensure that the programme responds adequately to changing local and national demands on elective care.^7^

There has been no independent evaluation of the HVLC surgical hubs policy published in peer-reviewed journals. We are aware of two ongoing, small-scale evaluations of the HVLC surgical hub programme: a qualitative study led by THIS Institute and a quantitative study led by the Health Foundation. We are working closely with these teams to ensure our efforts are collaborative and complementary. Barratt and colleagues conducted a mixed-method evaluation of the ‘Getting it Right First Time (GIRFT)’ programme in elective orthopaedic surgery^8^ and Briggs and colleagues describe a case report of four HVLC surgical hubs. Both studies report positive impacts across several outcomes, providing the rationale for scaling-up of the programme and for a more comprehensive evaluation.^9^ Our study (MEASURE) builds on this emerging, but small, evidence base, by providing a 4-year, independent, national evaluation of the HVLC surgical hub programme in England, commissioned by the National Institute for Health and Care Research.^10^

### Research question

What is the impact of the HVLC hubs on productivity, patient care and the workforce and what is the differential impact of different service models of HVLC surgical hubs?

A summary of the specific aims and objectives, work packages (WPs) and methods are provided in table 1.

**Table 1:** Research objectives, work packages

Research objectives	Work package(s)
To characterise the implementation timing, scale, scope and staffing of HVLC hubs currently working/being set-up in England.	1
To determine the impact of HVLC hubs on performance in terms of equity of access and uptake, and indicators including volume of activity, patients’ length of stay, waiting times and productivity across different patient populations.	2, 3, 4, 6
To explore the impact of HVLC models on professionals working in the hubs (including training, workload, skill-mix, turnover, absence, satisfaction, well-being and attitudes to and scope of practice).	3, 5
To explore the impact of HVLC hubs on the wider local NHS including spillovers in other areas of the NHS (eg, emergency care); workforce issues across the wider trust(s).	2, 3, 4, 5
To assess the impact of HVLC hubs on patients and carers (including views on travel/transport, nature/suitability, accessibility of premises and satisfaction, patient-reported outcome measures where available). The impact of hubs on the patient pathway, continuity of pre-surgical and post-surgical care and outpatient appointments will also be considered.	2, 3
To explore the implementation of HVLC hubs—how have changes been enacted and experienced and what are the barriers and facilitators to implementation.	1, 3
To compare resource utilisation and costs of care across different service models and typologies of HVLC hubs, conduct a cost-impact analysis and establish value for money.	1, 2, 6

HVLC, high-volume low-complexity ; NHS, National Health Service.

## Methods and analysis

### Study design

**Figure 1:**
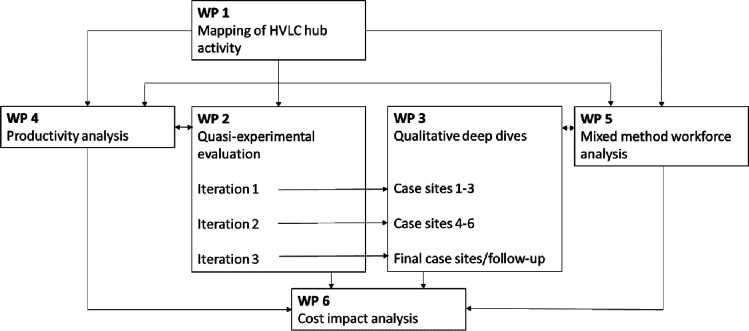
Study overview: the six integrated work packages of the MEASURE study. HVLC, high-volume low-complexity; WPs, work packages.

This mixed-methods study consists of six interlinked WPs (figure 1). Our study design is consistent with other recent large-scale evaluations of national health policies^8 11 12^ and our prior experience of undertaking national mixed-methods health policy evaluations has taught us that our study design must be flexible and able to adapt to shifting policy landscapes and priorities. Over the lifetime of our project, we anticipate changes in both the number of HVLC hubs that will be established and their service remit. We are mindful that the criteria for which patients are treated within hubs may be relaxed and/or expanded in response to changing influences on NHS demand. For example, some HVLC hubs are now inclusive of paediatrics. The individual WPs that are outlined below have been designed with this flexibility in mind to enable us to be responsive and to reflect evolving policy priorities.

### Theoretical framework

MEASURE will be informed by the Consolidated Framework for Implementation Research (CFIR),^13 14^ which has increasingly and successfully been applied to evaluate care model redesigns^15^ and to underpin mixed method^15^ and economic evaluations.^16^ The CFIR provides a comprehensive taxonomy of influencing factors that can be used to explore what works, where and why, across multiple contexts. The application of the CFIR to frame the MEASURE study is crucial for understanding the complexities surrounding the implementation of the HVLC programme and particularly for capturing the influence of local context on the programme’s implementation and impact. We will use the updated version of the CFIR, which in addition to the five core domains (intervention characteristics, wider context, local context, characteristics of the individuals involved and the process of implementation) includes innovation outcomes (eg, the ‘success or failure of innovation or HVLC hubs’).^14^

### Secondary data sources for quantitative analysis

Our quantitative analyses (WPs 1, 2, 4, 5, 6) will use NHS England Hospital Episode Statistics data and the National Joint Registry (NJR) in conjunction with various other additional data sources. Each data source, its use within the MEASURE project and its associated WP is outlined in table 2.

**Table 2:** Data sources and how they will be used in the MEASURE study

Data set	Use for MEASURE study	Work package
NHS England Hospital Episode Statistics data	Measuring processes and outcomes including volume of surgeries, waiting times, length of stay, readmission, emergency and acute care performance; risk classification and adjustment using the information on demographics, comorbidities and history of healthcare use.	2,4,6
National Joint Registry	Additional risk classification and stratification for orthopaedic patients using information on body mass index, American Society of Anesthesiologists (ASA) ASA score. Health outcomes including revision surgeries.	2, 4, 6
Civil registrations of deaths out of hospital (secondary cut)	Measuring mortality as an outcome.	2, 4, 6
Patient-reported outcome measures	Preoperative health and health outcomes for hip and knee replacement patients.	2, 4, 6
National Cost Collection	Measuring unit costs of elective care, and other care delivered by trusts.	4, 6
NHS workforce statistics, and trust annual accounts consolidation data (TACs)	NHS workforce statistics include monthly numbers of NHS Hospital staff working in trusts and other organisations. TACs include information on NHS Trusts income and expenditure items, received and incurred by type of inputs (labour, materials and capital). These data sets will be used to calculate, where possible, inputs used by surgical hubs.	4, 5, 6
Office for National Statistics (ONS) National Life Tables	Post-treatment life expectancy outcomes, to be used to quality adjust output produced by surgical hubs.	4
Model Health System’ data set	Hospital site-level efficiency measures such as theatre usage time.	2, 4, 6

NHS, National Health Service.

All patient data to be analysed are pseudonymised by NHS England or the NJR; we will not access any patient-identifiable information. All published outputs will include aggregated statistics, such as those at the level of national averages and organisation (ie, hospital trust and where possible surgical hub), with subgroup analysis at provider or commissioner levels or geographical areas or patient characteristics. No information will be published about individual patients or clinicians or that permits their identification.

### Work package 1: describe and classify current and planned models of HVLC hubs in England

To provide an accurate description of the HVLC surgical hubs programme three integrated phases of work will be conducted in parallel table 3. This will include describing funding, workforce, the date of commencement of any service change and current service configuration. GIRFT guidance identifies three distinct model types in England: standalone, integrated, ring-fenced.^7^ Intelligence obtained in this WP will be crucial for informing our subsequent quantitative and qualitative analyses. For example, dates of service change are essential for our quasi-experimental analysis (WP2) and model types and their impact on hub activity will be explored in-depth in WP2–4. This ‘live database’ will be continuously updated throughout the project to ensure our subsequent data collection and analyses are based on evolving hub implementation and reflect any anticipated changes to definitions of complexity and the changes in service remit of HVLC hubs more broadly.

**Table 3:** The three phases of planned work for work package 1

Phase	Data type	Output
1. Identifying characteristics of HVLC hubs	Desktop review of documentary evidence (eg, NHS/government publications and NHS board papers). Intelligence obtained through direct links to the HVLC team at Department of Health and Social Care and NHS England (eg, verbal communications and internal monitoring reports).	A regularly updated database of HVLC hub activity in England.
2. Data verification	Analysis of HES, NJR and Model Health System to explore and verify information about HVLC hubs identified in phase 1 and 3.	Verified information about the identification of low-complexity patients and the location of surgical hub sites in the data. Definitions of high/low complexity patients and how they can be identified in secondary data sets (to inform WP2 and WP4).
3. Qualitative service leader interviews	Qualitative interviews with 10–15 service leaders including: policy commissioners and those responsible for national delivery of HVLC hubs (eg, NHS England, Department of Health and Social Care (DHSC) and GIRFT representatives).	A rich contextual description of the development of the HVLC programme and its evolution over time. Hypotheses about the potential effects of HVLC hubs (WP2 and WP4) and identify potential case sites (WP3).

GIRFT, Getting it Right First Time; HES, Hospital Episode Statistics; HVLC, high-volume low-complexity ; NHS, National Health Service; NJR, National Joint Registry ; WP, work package.

### Work package 2: quantitative empirical evaluation of the effects of HVLC surgical hubs

We will perform a quasi-experimental evaluation of the HVLC hubs programme building on the MRC guidance on methods for the evaluation of complex interventions using observational data.^17^ In some cases, such as the 20 named new hubs mentioned in the government press release in August 2022^18^ we will have some certainty about when hospital trusts established HVLC surgical hubs and when they became operational. We will use information from the preliminary work and data verification in WP1 to clarify when changes to using a surgical hub model have occurred, and how these changes can be captured in our data sets.

We will use the variation in the timing of implementation of HVLC hubs across England using a staggered difference-in-difference methodology.^19^ By measuring the change in outcomes in areas when the HVLC hub model is adopted, and comparing these to the change in outcomes in (yet) unaffected areas, we will be able to estimate the intended and unintended effects of the HVLC hub model of care.

In instances where we cannot be sure of the timing of the opening surgical hubs or we cannot be sure that any ‘control’ areas are unaffected by the reform, we will explore other flexible empirical approaches such as cross-sectional analyses^20^ (which do not require variation over time), and event studies^21^ (which do not require unaffected control groups). We will also test whether different types of hubs, including stand-alone, integrated or ring-fenced hubs, achieve different outcomes.

We will use NHS England data and the NJR from financial years 2010/2011 through to the latest data available. The volume of low-complexity patients treated at hubs and at trusts associated with hubs is the primary outcome. Several secondary outcomes will also be examined including waiting times and length of stay, additional hospital-level productivity measures, health outcomes and further spillover effects on emergency and acute care. We will analyse all outcomes separately for high and low-complexity patients as defined by objective metrics (eg, a study by Protopapa *et al*).^22^ Throughout our analysis we will explore the consequences of the HVLC hubs programme for health inequalities by gender, age, ethnicity and socioeconomic status.

### Work package 3: in-depth, qualitative case studies of purposively selected HVLC hubs across England

We will conduct longitudinal, multimethod qualitative case studies at up to nine purposively-selected HVLC hubs across England. Our qualitative data collection will be conducted longitudinally alongside the three iterations of our quantitative evaluation (WP2 and WP4). Data collection will consist of: non-participant observational data, semi-structured interviews and documentary analysis.

A detailed overview of our planned qualitative work is provided in our separate qualitative study protocol (under review) which draws on published methodological work on designing and analysing ‘big qualitative studies’ in the context of national mixed methods policy evaluations.^23^ Central to our case-study design is the need for flexibility in our methods of sampling and data collection to enable us to be responsive to the policy landscape while balancing the need for breadth (a large and varied number of case-sites) and depth (exploring case-sites in sufficient detail to understand local context and policy adaptation).^23^

#### Purposive selection of qualitative case-sites

We will use each iteration of quantitative analysis (WP2 and WP4) to generate a list of potential qualitative case study sites. The study team and the Virtual Study Advisory Group (VSAG) (patients, clinical staff and NHS policymakers) will then work together to select case sites. We anticipate researchers spending 5–7 days at case sites over a 2-month period. Data collection will include approximately 12–15 hours of observation (eg, hub team meetings) and 10–15 interviews with staff (eg, anaesthetists, surgeons, nurses, hub administrators and local GIRFT coordinators, non-medical practitioners within extended surgical teams, surgical trainees and training programme directors), service leaders (eg, regional elective recovery leads) and patients and/or carers (sampled following INCLUDE principles) per site. Data collection will be informed by the CFIR resources^24^ and will focus on understanding: the implementation and impact of the HVLC programme, how hub provision fits with wider local surgical provision, workforce issues (WP5) and patient experience of referral and treatment at the hub.

Our analysis will be based on the ‘pen portrait’ approach,^25^ designed specifically for large, multidimensional qualitative data analysis—we will produce narrative descriptions (pen portraits) presented under the CFIR domains of each hub. Pen portrait data will then be analysed using reflexive thematic analysis.^26^ This interpretive^27^ analysis will allow detailed exploration within and across case-sites and will be tailored to address our research objectives for our main funder report and other additional qualitative outputs as appropriate.

### Work package 4

We will measure the productivity of the HVLC surgery hubs by means of two well-established measures: (1) the NHS productivity measure developed by the Centre for Health Economics^28^ and (2) the operational productivity measure developed by Lord Carter^29^ and used by NHS England in their Model Health System.^30^ Our productivity measures will use several different data sources as described in table 2. Our study period will start from the introduction of the HVLC hubs programme in 2021 to the latest year available.

We will analyse productivity at the level of each hub and within each clinical specialty, data permitting, therefore analysing productivity levels both within and between specialties. We propose to measure labour productivity, and if possible, the total factor productivity of the hubs. Productivity is calculated by comparing the total amount of healthcare output to input(s) for each HVLC hub. We will differentiate between types of surgical hub (stand-alone/ring-fenced/integrated) to investigate whether there are differences in terms of productivity levels achieved between types of surgical hubs. We will consult with the patient and public involvement (PPI) group early in the project to inform any refinements of productivity measures for hubs.

Further, the research team will investigate the potential impact of the establishment of HVLC surgery hubs on the wider system, flagging up any potential (unintended) effects on the productivity of other activity delivered by NHS Trusts. In particular, we will compare the measures of productivity within and between specialties, both for NHS Trusts with and without a hub.

We will adjust the output measure that feeds into the surgical hub productivity measure for changes in measures of quality (eg, survival, waiting time), with input from the PPI group and the VSAG to reach an agreement on proposed process and health outcome quality indicators (eg, survival, emergency readmissions, patient-reported outcome measures (PROMs)) to use in our analysis.

The operational productivity measure (Carter)^29^ will provide an alternative measure to cross-reference against the findings of the Centre for Health Economics (CHE) measure of productivity.

Finally, we will investigate the potential drivers of variations between surgical hubs’ productivity measures, if any are detected, following some of our previous work.^28 29^ The selection of determinants will be based on past literature, and the GIRFT ‘gateway frameworks’ metrics included in the Model Health System. Further determinants will be selected based on the specific set-ups of the surgical hubs, as informed by findings from WPs 1, 3 and 5, and as set out in the five domains of the CFIR. The five domains of the CFIR will also be used to help explain the findings of the analyses.

### Work package 5

The staffing of HVLC hubs, and the implications of changes in staffing for care delivered in wider trusts, are crucial contributors to the success of this policy. We will therefore seek to map the teams in place within trusts with longstanding and newly implemented HVLC hubs; identify wherever possible the staff working in the hubs and any changes in wider service staffing resulting from the introduction of newer hubs; and explore associations between different staffing models and the effectiveness of the HVLC hubs (as measured in WP2 and WP4).

Initially, we will explore existing data sources, including NHS Digital’s workforce statistics and data reflecting local labour markets, such as the wages component of the NHS market forces factor.^31^ We will request access to the NHS Electronic Staff Record (ESR) which would provide additional information but is not routinely accessible for research. We will examine workforce drivers of successful HVLC models by comparing hubs of different scales, team composition and staffing models based on outcome measures derived from WP2 and 4, taking account of the hierarchical nature of the data. We will monitor indicators of workforce well-being and sustainability such as rates of recruitment, retention, turnover, vacancies and use of bank and agency staff. Routinely collected staffing data may, however, be limited in detailing the exact size, composition and working practices of teams working across the hubs and wider NHS organisations. Therefore, we will build on WP1 to describe staffing models in place in the hubs, creating a taxonomy of these if there is variation in approaches between trusts and over time.

This will be coordinated with the primary qualitative data collection in WP3 where we will investigate staffing models in detail, taking into account the local context and the advice of our PPI panel and VSAG. Data collection from the case sites, summarised descriptively in the pen portraits will include workforce information, which will be explored in more depth. We will, for example, examine rotas, how teams in the HLVC hubs work, whether staff work across hubs and other NHS or private sector organisations and where health professionals moved from when the hubs were created. Within the qualitative case-studies, we will also gather data on broader labour market forces and the local contextual factors that impact on the workforce for each site, to explore how these external factors have influenced the nature and functioning of the hubs.

As part of the case study analysis, we will explore the impact of surgical hubs on staff training and development, for example, the number of surgical trainees in each hub, the extent to which trainees are embedded in the hub’s activity, perceptions of the quality of surgical training delivered within the hubs, training programmes for non-medical practitioners in the extended surgical team and any perceived unintended consequences in relation to training programmes. We will use CFIR to integrate and interpret the quantitative and qualitative data in a mixed-method analysis.

### Work package 6

We will conduct a cost-consequence analysis of the different HVLC models based on their estimated effects alongside estimated resource use, notably staff, equipment and capital investment. The estimated effects of HVLC models will follow from the data analysis on patient volumes, health outcomes and spillover effects estimated in WP2. Other consequences (eg, any impact on training practices) will be identified but may not be possible to measure or value meaningfully. Resource use will be derived from the documentation relating to staffing (workforce minimum data sets, and ESR) and capital investment applications,^32^ and by collating information across all WPs 1–5, alongside Personal Social Services Research Unit (PSSRU) unit cost estimates. We anticipate taking a ‘non-inferiority’ approach to the analysis of health outcomes, but if the quantitative analyses in WP2 identify a significant impact of the different HVLC models on patient health outcomes (eg, mortality, readmissions, PROMs), we will explore the feasibility of further health economic modelling.

### Mixed methods analysis to integrate qualitative and quantitative data across all work packages

Our separate quantitative and qualitative analyses will be jointly displayed in a mixed-methods matrix according to the relevant domains of the CFIR. This will enable a higher-level synthesis of our findings, that draws meta-inferences from both sources of data that are tailored to our overall study objectives and underpinned by relevant theory. This process of using implementation models to integrate large, complex quantitative and qualitative data has been successfully adopted in other large-scale policy evaluations.^11 12^

### Patient and public involvement

Given the complexity of our project and its duration, our PPI contributors are crucial for providing meaningful and active contributions to our study design and key decision-making. For instance, they will assist with the selection of quantitative outcomes and qualitative case study sites. We will speak with PPI contributors from a range of backgrounds, locations, genders and ages. They will include both carers and patients who are either on the waiting list for, or have undergone treatments that hubs are focusing on. We will also speak with PPI contributors based around our qualitative case study sites, who will help us to understand local context and services that may be affected by HVLC hubs to tailor our study materials, data collection, analysis and dissemination plans to their area.

## Ethics and dissemination

The study is registered through the research registry and qualitative components have been approved by the East Midlands-Nottingham Research Ethics Committee 23/EM/0231. The University of York is the study sponsor. Participants will provide informed consent for qualitative study components (WP 1, 3, 6). Routine care is not altered by the study and it does not raise significant ethical issues. Quantitative data analysis will involve secondary analysis of pseudonymised data sets available through a programme-level agreement between the Centre for Health Economics (University of York) and NHS England, and a separate data application to the NJR. Further data sets to be used in the quantitative analysis to inform resource use, labour, capital and intermediate inputs, will be the ESR, including pay-roll data and the trust account consolidation, that is, trusts financial accounts. Access to the Model Health System’ data set, NHS England, will also be sought, as it contains more granular data on theatre productivity, and resource use, including the staffing and skill-mix of surgical hubs.

The study commenced in May 2023 and is expected to run for 48 months.

Dissemination will include academic outputs (eg, conference presentations, peer-reviewed articles) and lay outputs (eg, policy briefs, project website, press releases and blog posts) disseminated through social media and our VSAG. We are in regular contact with HVLC programme leads at NHS England, through which (along with contacts at DHSC (Department of Health and Social Care), Royal College of Surgeons and GIRFT) we plan to foster a process of live-feedback throughout the project to ensure our findings and study design is considerate of and influences policy development. This is our most direct route to impact.

## Supplementary Material

Reviewer comments

Author's manuscript
