# Supplementary material for: Mixed Methods EvAluation of the high-volume low-complexity Surgical hUb pRogrammE (MEASURE): a mixed methods study protocol
Source: BMJ Open. 2024 Apr 19;14(4):e086338. doi: 10.1136/bmjopen-2024-086338 (PMC11033628; doi:10.1136/bmjopen-2024-086338)
Supplement: Author's manuscript [file bmjopen-2024-086338.draft_revisions.pdf]

BMJ Open is committed to open peer review. As part of this commitment we make the peer review history of every article we publish publicly available.

When an article is published we post the peer reviewers' comments and the authors' responses online. We also post the versions of the paper that were used during peer review. These are the versions that the peer review comments apply to.

The versions of the paper that follow are the versions that were submitted during the peer review process. They are not the versions of record or the final published versions. They should not be cited or distributed as the published version of this manuscript.

BMJ Open is an open access journal and the full, final, typeset and author-corrected version of record of the manuscript is available on our site with no access controls, subscription charges or pay-per-view fees (http://bmjopen.bmj.com).

If you have any questions on BMJ Open's open peer review process please email info.bmjopen@bmj.com

BMJ Open

Mixed Methods EvAluation of the high-volume low-complexity Surgical hUb pRogrammE (MEASURE): a mixed methods study protocol

Journal:	*BMJ Open*
Manuscript ID	bmjopen-2024-086338
Article Type:	Protocol
Date Submitted by the Author:	13-Mar-2024

Complete List of Authors:	Scantlebury, Arabella; University of York, York Trials Unit, Department of Health Sciences<br>Sivey, Peter; University of York, Centre for Health Economics<br>Anteneh, Zecharias; University of York, Centre for Health Economics<br>Davies, Simon; Hull York Medical School, Centre for Health and Population Sciences<br>Glerum-Brooks, Karen; University of York, York Trials Unit<br>Lampard, Pete; University of York, Department of Health Sciences<br>Saad, Ahmed; James Cook University Hospital, Opthamology; Hull York Medical School<br>Wen, Jinglin; University of York, Centre for Health Economics<br>Ayres, Ben; St George's University Hospitals NHS Foundation Trust<br>Bloor, Karen; University of York, Department of Health Sciences<br>Castelli , Adriana; University of York, Centre for Health Economics<br>Castro-Avila, Ana; University of York, Health Sciences<br>Davies, Firoza; University of York<br>Gutacker, Nils; University of York, Centre for Health Economics<br>Rangan, Amar; James Cook University Hospital, Trauma and Orthopaedics; University of York, Department of Health Sciences<br>Street, A; LSE<br>Adamson, Joy; University of York, Health Sciences

Keywords:	HEALTH ECONOMICS, Health policy < HEALTH SERVICES ADMINISTRATION & MANAGEMENT, Protocols & guidelines < HEALTH SERVICES ADMINISTRATION & MANAGEMENT, ORTHOPAEDIC & TRAUMA SURGERY, QUALITATIVE RESEARCH, SURGERY

SCHOLARONE™
Manuscripts

Mixed Methods EvAluation of the high-volume low-complexity Surgical hUb pRogrammE (MEASURE): a mixed methods study protocol

Authors names: Arabella Scantlebury (0000-0003-3518-2740), Peter Sivey (0000-0002-3703-615X), Zecharias Anteneh (0000-0001-6588-9056), Benjamin Ayres (0000-0002-8738-7724), Karen Bloor (0000-0003-4852-9854), Adriana Castelli (0000-0002-2546-419X), Ana Castro Avila (0000-0003-4475-4325), Firoza Davies, Simon Davies (0000-0003-2786-6237), Karen Glerum-Brooks (0000-0001-7871-6293), Nils Gutacker (0000-0002-2833-0621), Pete Lampard (0000-0003-3963-8602), Amar Rangan (0000-0002-5452-8578), Ahmed Saad (0000-0002-5159-8001), Andrew Street (0000-0002-2540-0364), Jinglin Wen (0000-0002-2575-8497), Joy Adamson (0000-0002-9860-0850).

York Trials Unit, Department of Health Sciences, ARRC Building, University of York, York, YO10 5DD, UK, Assistant Professor, Arabella Scantlebury

Centre for Health Economics, University of York, YO10 5DD, UK, Reader, Peter Sivey

Department of Health Sciences, ARRC Building, University of York, York, YO10 5DD, UK,

Centre for Health Economics, University of York, York, YO10 5DD, UK, Research Fellow, Zecharias Anteneh

St George's University Hospitals NHS Trust, London, SW17 0QT, UK, Consultant Urologist, Ben Ayres

Department of Health Sciences, ARRC Building, University of York, York, YO10 5DD, UK, Professor, Karen Bloor

For peer review only

Centre for Health Economics, University of York, YO10 5DD, UK, Senior Research Fellow, Adriana Castelli

Department of Health Sciences, ARRC Building, University of York, York, YO10 5DD, UK, Research Fellow, Ana Castro-Avila

York Trials Unit, Department of Health Sciences, ARRC Building, University of York, York, YO10 5DD, UK, Lay representative, Firoza Davies,

Centre for Health and Population Sciences, Hull York Medical School, Siwards Way, Heslington, York, YO10 5DD, UK, Reader in Perioperative Medicine, Consultant Anaesthetist, Simon Davies

York Trials Unit, Department of Health Sciences, ARRC Building, University of York, York, YO10 5DD, UK, Patient and Public Involvement and Stakeholder Engagement Manager, Karen Glerum Brooks,

Centre for Health Economics, University of York, YO10 5DD, UK, Professor, Nils Gutacker

Department of Health Sciences, ARRC Building, University of York, York, YO10 5DD, UK, Research Fellow, Pete Lampard

York Trials Unit, Department of Health Sciences, ARRC Building, University of York, York, YO10 5DD, UK, Research Fellow, and South Tees Hospital NHS Foundation Trust, The James Cook University Hospital, Middlesbrough, TS4 3BW, UK. Professor and Consultant Orthopaedic Surgeon Amar Rangan.

South Tees Hospital NHS Foundation Trust, The James Cook University Hospital, Middlesbrough, TS4 3BW, UK, Hull York Medical School, Siwards Way, Heslington, York, YO10 5DD, UK, and Zagazig University, Zagazig, P.O Box s44519, Egypt, Consultant and Senior Clinical Lecturer, Ahmed Saad

Department of Health Policy, London School of Economics, London, WC2A 2AE, Professor, Andrew Street

For peer review only - http://bmjopen.bmj.com/site/about/guidelines.xhtml

Centre for Health Economics, University of York, YO10 5DD, UK, Research Fellow, Jinglin Wen

York Trials Unit, Department of Health Sciences, ARRC Building, University of York, York, YO10 5DD, UK, Professor, Joy Adamson

Correspondence to: Arabella Scantlebury, York Trials Unit, Department of Health Sciences, University of York, ARRC Building, University of York, York, YO10 5 DD, UK.

For peer review only

For peer review only - http://bmjopen.bmj.com/site/about/guidelines.xhtml

ABSTRACT

INTRODUCTION: The waiting list for elective surgery in England recently reached over 7.8 million people and waiting time targets have been missed since 2010. The high-volume low complexity (HVLC) surgical hubs programme aims to tackle the backlog of patients awaiting elective surgery treatment in England. This study will evaluate the impact of HVLC surgical hubs on productivity, patient care and the workforce.

METHODS AND ANALYSIS: This 4-year project consists of 6 inter-linked work packages (WPs) and is informed by the Consolidated Framework for Implementation Research. *WP1*: Mapping current and future HVLC provision in England through document analysis, quantitative datasets (e.g., Hospital Episodes Statistics) and interviews with national service leaders. *WP2*: Exploring the effects of HVLC hubs on key performance outcomes, primarily the volume of low-complexity patients treated, using quasi-experimental methods. *WP3*: Exploring the impact and implementation of HVLC hubs on patients, health professionals and the local NHS through approximately nine longitudinal, multi-method qualitative case studies. *WP4*: Assessing productivity of HVLC surgical hubs using the Centre for Health Economics NHS productivity measure and Lord Carter's operational productivity measure. *WP5*: Conducting a mixed-methods appraisal will assess the influence of HVLC surgical hubs on the workforce using: qualitative data (WP3) and quantitative data (e.g. NHS England's workforce statistics and intelligence from WP2). *WP6*: Analysing the costs and consequences of HVLC surgical hubs will assess their achievements in relation to their resource use to establish value for money. A Patient and Public Involvement Group (PPI) will contribute to study design and materials.

ETHICS AND DISSEMINATION: The study has been approved by the East Midlands – Nottingham Research Ethics Committee 23/EM/0231. Dissemination plans include multiple academic and non-

For peer review only

academic outputs (e.g. Peer-reviewed journals, conferences, social media) and a continuous, feedback-loop of findings to key stakeholders (e.g. NHS England) to influence policy development.

STUDY REGISTRATION: Researchregistry9364.

STRENGTHS AND LIMITATIONS

- The largest, independent, longitudinal, mixed-methods study of the High-Volume Low Complexity (HVLC) surgical hub programme in England.
- Aims to identify the impact of HVLC surgical hubs on outcomes, productivity, patient care, and the workforce and the differential impact of different service models of HVLC surgical hubs
- Our flexible study design and application of implementation theory will ensure our findings capture local context and are relevant to current policy.
- Quantitative analysis will be constrained by the quantity and quality of available information about the implementation and management of HVLC surgical hubs.
- Models of HVLC surgical hubs are expected to be locally tailored and the policy landscape is complex and shifting. This may present a challenge for analysis.

INTRODUCTION

Globally, health services are experiencing unprecedented levels of demand and are struggling to recover from the effects of the COVID-19 pandemic.(1-4) During 2020, approximately 34% of elective care was cancelled in the UK with elective hip replacements falling to almost zero between March and April 2020.(1, 5) The pandemic came against a back-drop of worsening performance against England's 18-week target for elective surgery since 2011,(6) and improvement post-pandemic has been variable.

NHS England has launched an elective recovery plan (2) designed specifically to address the "backlog" of patients awaiting routine surgical treatment and to increase elective throughput of the NHS to 130%

For peer review only - http://bmjopen.bmj.com/site/about/guidelines.xhtml

of pre-pandemic "business as usual" volume by 2024/2025.(2) The high-volume, low-complexity (HVLC) surgical hubs model is part of NHS England's elective recovery blueprint, and has been specifically designed to reduce waiting lists for elective surgery in England. By ring-fencing staff and facilities in designated locations, the HVLC surgical hub programme aims to improve efficiency and productivity in the elective pathway by focussing efforts exclusively on treating low complexity elective patients more quickly. This contrasts with the usual model of elective care where patients with less complex conditions are treated in acute hospitals where surgeons, anaesthetists, nursing and diagnostic staff have competing demands from more complex cases and patients requiring urgent and emergency care.(7)

As of February 2024, NHS England reported that there were over 90 HVLC surgical hubs nationally. This includes "long-standing" HVLC hubs that have been operational since before the official launch of the programme in 2022 and "new" surgical hubs established subsequently. In 2023, the Royal College of Surgeons in collaboration with the Royal College of Anaesthetists developed a non-mandatory accreditation scheme, which aims to ensure national standards for HVLC surgical hubs, with 31 hubs receiving as of January 2024. Plans for the HVLC programme continue to evolve. It is expected that in addition to further HVLC surgical hubs being set-up and accredited that the type of patients directed to HVLC surgical hubs for treatment will expand. This evolution is designed to ensure that the programme responds adequately to changing local and national demands on elective care.(7)

There has been no independent evaluation of the HVLC surgical hubs policy published in peer-reviewed journals. We are aware of two on-going, small-scale evaluations of the HVLC surgical hub programme: a qualitative study led by THIS Institute and a quantitative study led by the Health Foundation. We are working closely with these teams to ensure our efforts are collaborative and complementary. Barratt and colleagues conducted a mixed-method evaluation of the "Getting it Right First Time (GIRFT) programme in elective orthopaedic surgery (8) and Briggs and colleagues describe a case report of four HVLC surgical hubs. Both studies report positive impacts across several outcomes,

For peer review only

providing the rationale for scaling-up of the programme and for a more comprehensive evaluation. (9) Our study (MEASURE) builds on this emerging, but small, evidence base, by providing a four-year, independent, national evaluation of the HVLC surgical hub programme in England, commissioned by the National Institute for Health and Care Research (NIHR).(10)

Research question

What is the impact of the HVLC hubs on productivity, patient care, and the workforce and what is the differential impact of different service models of HVLC surgical hubs?

A summary of the specific aims and objectives, WPs and methods are provided in table 1.

Table 1 Research objectives, work packages

**Research objectives**	Work Package(s)
To characterise the implementation timing, scale, scope and staffing of HVLC hubs currently working/being set up in England	1
To determine the impact of HVLC hubs on performance in terms of equity of access and uptake, and indicators including volume of activity, patients' length of stay, waiting times, and productivity across different patient populations	2, 3, 4, 6
To explore the impact of HVLC models on professionals working in the hubs (including training, workload, skill-mix, turnover, absence, satisfaction, well-being and attitudes to and scope of practice)	3, 5
To explore the impact of HVLC hubs on the wider local NHS including spillovers in other areas of the NHS (e.g. emergency care); workforce issues across the wider trust(s)	2, 3, 4, 5
To assess the impact of HVLC hubs on patients and carers (including views on travel/transport, nature/suitability, accessibility of premises, and satisfaction, patient reported outcome measures (PROMs) where available). The impact of hubs on the patient pathway, continuity of pre- and post-surgical care, and outpatient appointments will also be considered	2, 3
To explore the implementation of HVLC hubs – how have changes been enacted and experienced and what are the barriers and facilitators to implementation	1, 3
To compare resource utilisation and costs of care across different service models and typologies of HVLC hubs, conduct a cost-impact analysis and establish value for money	1, 2, 6

For peer review only - http://bmjopen.bmj.com/site/about/guidelines.xhtml

METHODS

Study design

This mixed-methods study consists of six inter-linked work packages (WPs) (Figure 1). Our study design is consistent with other recent large-scale evaluations of national health policies (8, 11, 12) and our prior experience of undertaking national mixed-methods health policy evaluations has taught us that our study design must be flexible and able to adapt to shifting policy landscapes and priorities. Over the lifetime of our project, we anticipate changes in both the number of HVLC hubs that will be established and their service remit. We are mindful that the criteria for which patients are treated within hubs may be relaxed and/or expanded in response to changing influences on NHS demand. For example, some HVLC hubs are now inclusive of paediatrics. The individual work packages that are outlined below have been designed with this flexibility in mind to enable us to be responsive and to reflect evolving policy priorities.

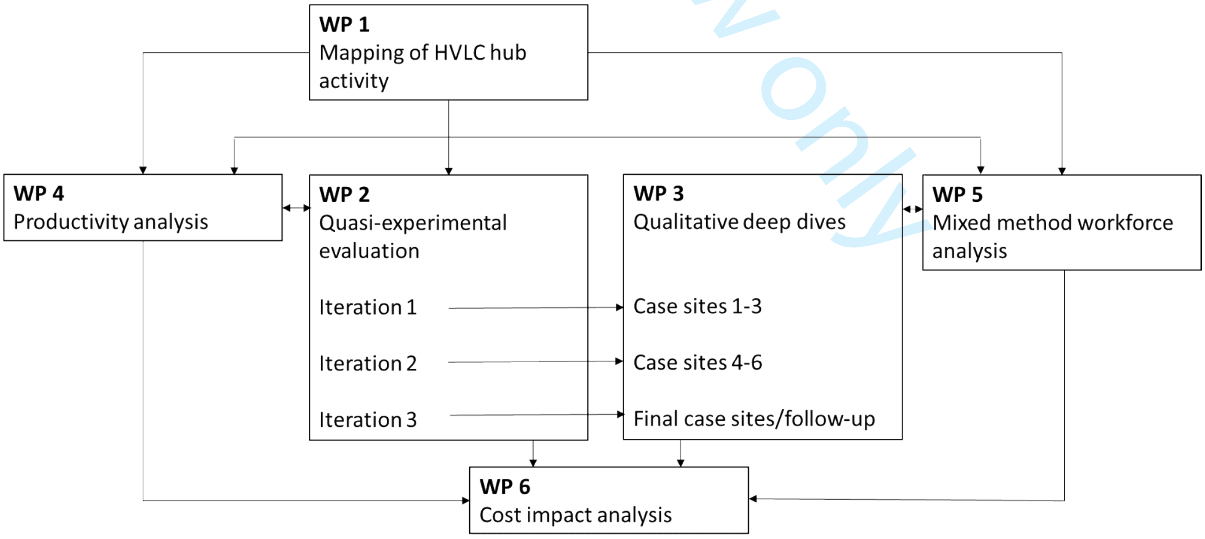

Figure 1 The Six integrated Work Packages of the MEASURE study

For peer review only

For peer review only - http://bmjopen.bmj.com/site/about/guidelines.xhtml

Theoretical framework

MEASURE will be informed by the Consolidated Framework for Implementation Research (CFIR),(13, 14) which has increasingly and successfully been applied to evaluate care model redesigns (15) and to underpin mixed method(15) and economic evaluations.(16) The CFIR provides a comprehensive taxonomy of influencing factors that can be used to explore what works, where and why, across multiple contexts. The application of the CFIR to frame the MEASURE study is crucial for understanding the complexities surrounding the implementation of the HVLC programme and particularly for capturing the influence of local context on the programme's implementation and impact. We will use the updated version of the CFIR, which in addition to the 5 core domains (intervention characteristics, wider context, local context, characteristics of the individuals involved and the process of implementation) includes innovation outcomes (e.g. the 'success or failure of innovation or HVLC hubs').(14)

Secondary data sources for quantitative analysis

Our quantitative analyses (WPs 1, 2, 4, 5, 6) will use NHS England Hospital Episode Statistics (HES) data and the National Joint Registry (NJR) in conjunction with various other additional data sources. Each data source, its use within the MEASURE project and associated work package is outlined in table 2.

All patient data to be analysed are pseudonymised by NHS England or the National Joint Registry (NJR); we will not access any patient-identifiable information. All published outputs will include aggregated statistics, such as those at the level of national averages and organisation (i.e. hospital trust and where possible surgical hub), with subgroup analysis at provider or commissioner levels or geographical areas or patient characteristics. No information will be published about individual patients or clinicians or that permits their identification.

Table 2. Data sources and how they will be used in the MEASURE study.

For peer review only

Data set	Use for MEASURE study	Work Package
NHS England Hospital Episode Statistics (HES) data.	Measuring processes and outcomes including volume of surgeries, waiting times, length of stay, readmission, emergency and acute care performance; risk classification and adjustment using information on demographics, co-morbidities and history of health care use.	2,4,6
National Joint Registry	Additional risk classification and stratification for orthopaedic patients using information on Body Mass Index (BMI), American Society of Anaesthesiologists (ASA) ASA score. Health outcomes including revision surgeries.	2, 4, 6
Civil Registrations of deaths out of hospital (Secondary Cut),	Measuring mortality as an outcome.	2, 4, 6
Patient Reported Outcome Measures (PROMs)	Pre-operative health and health outcomes for hip and knee replacement patients.	2, 4, 6
National Cost Collection	Measuring unit costs of elective care, and other care delivered by Trusts	4, 6
NHS workforce statistics, and Trust annual accounts consolidation data (TACs)	NHS workforce statistics include monthly numbers of NHS Hospital staff working in Trusts and other organisations. TACs include information on NHS Trusts income and expenditure items, received and incurred by type of inputs (labour, materials and capital). These datasets will be used to calculate, where possible, inputs used by surgical hubs.	4, 5, 6
ONS National Life Tables	Post-treatment life expectancy outcomes, to be used to quality adjust output produced by surgical hubs	4
Model Health System' dataset	Hospital site-level efficiency measures such as theatre usage time.	2, 4, 6

181

For peer review only - http://bmjopen.bmj.com/site/about/guidelines.xhtml

182 ## Work Package 1- describe and classify current and planned models of HVLC hubs in England

184 To provide an accurate description of the HVLC surgical hubs programme three integrated phases of work will be conducted in parallel (table 3). This will include describing funding, workforce, the date of commencement of any service change and current service configuration. GIRFT guidance identifies three distinct model types in England: standalone, integrated, ring-fenced.(7) Intelligence obtained in this work package will be crucial for informing our subsequent quantitative and qualitative analyses. For example, dates of service change are essential for our quasi-experimental analysis (WP2) and model types and their impact on hub activity will be explored in-depth in WP2-4. This "live database" will be continuously updated throughout the project to ensure our subsequent data collection and analyses are based on evolving hub implementation and reflects any anticipated changes to definitions of complexity and the changes in service remit of HVLC hubs more broadly.

194 *Table 3 The three phases of planned work for Work Package 1*

Phase	Data type	Output
1: Identifying characteristics of HVLC hubs	Desktop review of documentary evidence (e.g. NHS/government publications and NHS board papers).<br><br>Intelligence obtained through direct links to the HVLC team at department of Health and Social Care and NHS England (e.g. verbal communications and internal monitoring reports).	A regularly updated database of HVLC hub activity in England.
2: Data Verification	Analysis of HES, NJR and Model Health System to explore and verify information about HVLC hubs identified in phase 1 and 3.	Verified information about identification of low-complexity patients and location of surgical hub sites in the data.<br>Definitions of high/low complexity patients and how they can be identified in secondary data sets (to inform WP2 and WP4).

For peer review only - http://bmjopen.bmj.com/site/about/guidelines.xhtml

3. Qualitative service leader interviews	Qualitative interviews with 10-15 service leaders including: policy commissioners and those responsible for national delivery of HVLC hubs (e.g. NHS England, DHSC and GIRFT representatives).	A rich contextual description of the development of the HVLC programme and its evolution over time. Hypotheses about the potential effects of HVLC hubs (WP2 and WP4) and identify potential case sites (WP3).

Work Package 2 – quantitative empirical evaluation of the effects of HVLC surgical hubs

We will perform a quasi-experimental evaluation of the HVLC hubs programme building on the MRC guidance on methods for the evaluation of complex interventions using observational data.(17) In some cases, such as the 20 named new hubs mentioned in the government press release in August 2022(18)we will have some certainty about when hospital trusts established HVLC surgical hubs and when they became operational. We will use information from the preliminary work and data verification in WP1 to clarify when changes to using a surgical hub model have occurred, and how these changes can be captured in our datasets.

We will use the variation in timing of implementation of HVLC hubs across England using a staggered difference-in-difference methodology.(19) By measuring the change in outcomes in areas when the HVLC hub model is adopted, and comparing these to the change in outcomes in (yet) unaffected areas, we will be able to estimate the intended and unintended effects of the HVLC hub model of care.

In instances where we cannot be sure of the timing of the opening surgical hubs or we cannot be sure that any ‘control’ areas are unaffected by the reform, we will explore other flexible empirical approaches such as cross-sectional analyses (20)(which do not require variation over time), and event studies(21) (which do not require unaffected control groups). We will also test whether different types of hubs, including stand-alone, integrated or ring-fenced hubs, achieve different outcomes.

We will use NHS England data and the NJR from financial years 2010/11 through to the latest data available. The volume of low-complexity patients treated at hubs and at trusts associated with hubs is the primary outcome. Several secondary outcomes will also be examined including waiting times and length of stay (LOS), additional hospital-level productivity measures, health outcomes and further

For peer review only

For peer review only - http://bmjopen.bmj.com/site/about/guidelines.xhtml

spillover effects on emergency and acute care. We will analyse all outcomes separately for high and low-complexity patients as defined by objective metrics (eg (22)). Throughout our analysis we will explore the consequences of the HVLC hubs programme for health inequalities by gender, age, ethnicity and socio-economic status.

Work Package 3 – in-depth, qualitative case studies of purposively selected HVLC hubs across England

We will conduct longitudinal, multi-method qualitative case studies at up to 9 purposively-selected HVLC hubs across England. Our qualitative data collection will be conducted longitudinally alongside the three iterations of our quantitative evaluation (WP2 and WP4). Data collection will consist of: non-participant observational data, semi-structured interviews and documentary analysis.

A detailed overview of our planned qualitative work is provided in our separate qualitative study protocol- (under review) which draws upon published methodological work on designing and analysing "big qualitative studies" in the context of national mixed methods policy evaluations.(23) Central to our case-study design is the need for flexibility in our methods of sampling and data collection to enable us to be responsive to the policy landscape whilst balancing the need for breadth (a large and varied number of case-sites) and depth (exploring case-sites in sufficient detail to understand local context and policy adaptation).(23)

Purposive selection of qualitative case-sites

We will use each iteration of quantitative analysis (WP2 and WP4) to generate a list of potential qualitative case study sites. The study team and the Virtual Study Advisory Group (VSAG) (patients, clinical staff and NHS policymakers) will then work together to select case sites. We anticipate researchers spending 5-7 days at case sites over a two-month period. Data collection will include approximately 12-15 hours of observation (e.g., hub team meetings) and 10-15 interviews with staff (e.g. anaesthetists, surgeons, nurses, hub administrators and local GIRFT co-ordinators, non-medical

For peer review only - http://bmjopen.bmj.com/site/about/guidelines.xhtml

practiioners within extended surgical teams, surgical trainees and training programme directors), service leaders (e.g. regional elective recovery leads) and patients and/or carers (sampled following INCLUDE principles) per site. Data collection will be informed by the CFIR resources(24) and will focus on understanding: the implementation and impact of the HVLC programme, how hub provision fits with wider local surgical provision, workforce issues (WP5) and patient experience of referral and treatment at the hub.

Our analysis will be based on the "pen portrait" approach,(25) designed specifically for large, multi-dimensional qualitative data analysis – we will produce narrative descriptions (pen portraits) presented under the CFIR domains of each hub. Pen portrait data will then be analysed using reflexive thematic analysis.(26) This interpretive(27) analysis will allow detailed exploration within and across case-sites and will be tailored to address our research objectives for our main funder report and other additional qualitative outputs as appropriate.

Work Package 4

We will measure the productivity of the HVLC surgery hubs by means of two well-established measures: (i) the NHS productivity measure developed by the Centre for Health Economics (28)and (ii) the operational productivity measure developed by Lord Carter(29) and used by NHS England in their Model Health System.(30) Our productivity measures will use several different data sources as described in Table 2. Our study period will start from the introduction of the HVLC hubs programme in 2021 to the latest year available.

We will analyse productivity at the level of each hub and within each clinical specialty, data permitting, therefore analysing productivity levels both within and between specialties. We propose to measure labour productivity, and if possible, total factor productivity of the hubs. Productivity is calculated by comparing the total amount of healthcare output to input(s) for each HVLC hub. We will differentiate between types of surgical hub (stand-alone/ring-fenced/integrated) to investigate whether there are

For peer review only - http://bmjopen.bmj.com/site/about/guidelines.xhtml

differences in terms of productivity levels achieved between types of surgical hubs. We will consult with the PPI group early in the project to inform any refinements of productivity measures for hubs.

Further, the research team will investigate the potential impact of the establishment of HVLC surgery hubs on the wider system, flagging up any potential (unintended) effects on the productivity of other activity delivered by NHS Trusts. In particular, we will compare the measures of productivity within and between specialty, both for NHS Trusts with and without a hub.

We will adjust the output measure that feeds into the surgical hub productivity measure for changes in measures of quality (e.g. survival, waiting time), with input from the PPI group and the VSAG to reach an agreement on proposed process and health outcome quality indicators (e.g., survival, emergency readmissions, PROMs) to use in our analysis.

The operational productivity measure (Carter)(29) will provide an alternative measure to cross-reference against the findings of the CHE measure of productivity.

Finally, we will investigate the potential drivers of variations between surgical hubs' productivity measures, if any are detected, following some of our previous work.(28, 29) The selection of determinants will be based on past literature, and the GIRFT 'gateway frameworks' metrics included in the Model Health System. Further determinants will be selected based on the specific set-ups of the surgical hubs, as informed by findings from WPs 1, 3, and 5, and as set out in the five domains of the CFIR. The five domains of the CFIR will also be used to help explain findings of the analyses.

Work Package 5

The staffing of HVLC hubs, and the implications of changes in staffing for care delivered in wider Trusts, are crucial contributors to the success of this policy. We will therefore seek to map the teams in place within Trusts with longstanding and newly implemented HVLC hubs; identify wherever possible the staff working in the hubs and any changes in wider service staffing resulting from the introduction of

For peer review only - http://bmjopen.bmj.com/site/about/guidelines.xhtml

newer hubs; and explore associations between different staffing models and the effectiveness of the HVLC hubs (as measured in WP2 and WP4).

Initially, we will explore existing data sources, including NHS Digital's workforce statistics and data reflecting local labour markets, such as the wages component of the NHS market forces factor.(31) We will request access to the NHS Electronic Staff Record (ESR) which would provide additional information but is not routinely accessible for research. We will examine workforce drivers of successful HVLC models by comparing hubs of different scale, team composition and staffing models based on outcome measures derived from WP2 and 4, taking account of the hierarchical nature of the data. We will monitor indicators of workforce wellbeing and sustainability such as rates of recruitment, retention, turnover, vacancies and use of bank and agency staff. Routinely collected staffing data may, however, be limited in detailing the exact size, composition and working practices of teams working across the hubs and wider NHS organisations. Therefore, we will build on WP1 to describe staffing models in place in the hubs, creating a taxonomy of these if there is variation in approaches between Trusts and over time.

This will be coordinated with the primary qualitative data collection in WP3 where we will investigate staffing models in detail, taking into account the local context and the advice of our PPI panel and VSAG. Data collection from the case sites, summarised descriptively in the pen portraits will include workforce information, which will be explored in more depth. We will, for example, examine rotas, how teams in the HLVC hubs work, whether staff work across hubs and other NHS or private sector organisations and where health professionals moved from when the hubs were created. Within the qualitative case-studies, we will also gather data on broader labour market forces and the local contextual factors that impact on workforce for each site, to explore how these external factors have influenced the nature and functioning of the hubs.

As part of the case study analysis, we will explore the impact of surgical hubs on staff training and development, for example, the number of surgical trainees in each hub, the extent to which trainees

For peer review only - http://bmjopen.bmj.com/site/about/guidelines.xhtml

are embedded in the hub's activity, perceptions of the quality of surgical training delivered within the hubs, training programmes for non-medical practitioners in the extended surgical team and any perceived unintended consequences in relation to training programmes. We will use CFIR to integrate and interpret the quantitative and qualitative data in a mixed-method analysis.

Work Package 6

We will conduct a cost-consequence analysis of the different HVLC models based on their estimated effects alongside estimated resource use, notably staff, equipment and capital investment. The estimated effects of HVLC models will follow from the data analysis on patient volumes, health outcomes and spillover effects estimated in WP2. Other consequences (e.g. any impact on training practices) will be identified but may not be possible to measure or value meaningfully. Resource use will be derived from the documentation relating to staffing (workforce minimum datasets, and ESR) and capital investment applications,(32) and by collating information across all WPs 1-5, alongside PSSRU unit cost estimates. We anticipate taking a 'non-inferiority' approach to analysis of health outcomes, but if the quantitative analyses in WP2 identify a significant impact of the different HVLC models on patient health outcomes (e.g. mortality, readmissions, PROMS), we will explore the feasibility of further health economic modelling.

Mixed methods analysis to integrate qualitative and quantitative data across all work packages

Our separate quantitative and qualitative analyses will be jointly displayed in a mixed-methods matrix according to the relevant domains of the CFIR. This will enable a higher-level synthesis of our findings, that draws meta-inferences from both sources of data that are tailored to our overall study objectives and underpinned by relevant theory. This process of using implementation models to integrate large,

For peer review only

complex quantitative and qualitative data has been successfully adopted in other large-scale policy evaluations.(11, 12)

Patient and Public Involvement Statement

Given the complexity of our project and its duration, our PPI contributors are crucial for providing meaningful and active contributions to our study design and key decision-making. For instance, they will assist with the selection of quantitative outcomes and qualitative case study sites. We will speak with PPI contributors from a range of backgrounds, locations, genders, ages. They will include both carers and patients who are either on the waiting list for, or have undergone treatments that hubs are focusing on. We will also speak with PPI contributors based around our qualitative case study sites, who will help us to understand local context and services which may be affected by HVLC hubs to tailor our study materials, data collection, analysis and dissemination plans to their area.

Ethics and Dissemination

The study is registered through the research registry and qualitative components have been approved by East Midlands-Nottingham Research Ethics Committee 23/EM/0231. University of York are the study sponsor. Participants will provide informed consent for qualitative study components (WP 1, 3, 6). Routine care is not altered by the study and it does not raise significant ethical issues. Quantitative data analysis will involve secondary analysis of pseudonymised datasets available through a programme-level agreement between the Centre for Health Economics (University of York) and NHS England, and a separate data application to the National Joint Registry. Further datasets to be used in the quantitative analysis to inform resource use, labour, capital, and intermediate inputs, will be the Electronic Staff Records (ESR), including pay-roll data, and the Trust Account Consolidation (TACs), i.e. Trusts financial accounts. Access to the Model Health System' dataset, NHS England, will also be sought, as it contains more granular data on theatre productivity, and resource use, including the staffing and skill-mix of surgical hubs.

For peer review only - http://bmjopen.bmj.com/site/about/guidelines.xhtml

The study commenced in May 2023 and is expected to run for 48 months.

Dissemination will include academic outputs (e.g., conference presentations, peer-reviewed articles) and lay outputs (e.g., policy briefs, project website, press releases and blog posts) disseminated through social media and our VSAG. We are in regular contact with HVLC programme leads at NHS England, through which (along with contacts at DHSC, Royal College of Surgeons and GIRFT) we plan to foster a process of live-feedback throughout the project to ensure our findings and study design is considerate of and influences policy development. This is our most direct route to impact.

ACKNOWLEDGEMENTS

We would like to thank our public contributors, Ms Elena Bechberger and Professor Tim Briggs for their support and collaboration with the MEASURE study.

AUTHORS CONTRIBUTIONS

JA and PS are the study's Co-Chief Investigators. JA, PS, AC, AS, KB are work package leads. The research question was developed and the study was designed by: JA, KB, AC, SD, KG-B, NG, AR, PS, ASc, ASt. ASc drafted the manuscript. The protocol was developed and refined by: JA, BA, ZA, KB, AC, AC-A, SD, KG-B, NG, PL, AR, PS, ASc, ASt, JW. All authors have reviewed, revised and approved the final manuscript.

FUNDING STATEMENT

This work was supported by the National Institute for Health Research, Health Services and Delivery Research Programme (NIHR HS&DR), Grant number: NIHR153387. The views expressed are those of the authors and not necessarily those of the NIHR or the Department of Health and Social Care.

For peer review only

For peer review only - http://bmjopen.bmj.com/site/about/guidelines.xhtml

ETHICS APPROVAL

Approval from the study has been obtained from the Health Research Authority. The protocol was reviewed and received a favourable opinion from the National Health Service (NHS) East Midlands-Nottingham Research Ethics Committee REC: 23/EM/0231.

COMPETING INTERESTS STATEMENT

BA, is the Clinical Director of Urology for NHS London and has been involved in the initial implementation of the HVLC surgical hubs in London. The authors declare no other competing interests.

REFERENCES

1. Burn S, Propper, C., Stoye, G., Warner, M., Aylin, P., Bottle., A. What happened to English NHS Hospital activity during the COVID-19 pandemic? : IFS Briefing Note. BN328; 2021 [
2. England N. Delivery plan for tacking the covid-19 backlog of elective care. . 2022a.
3. England. N. Guide to NHS Waiting times in England. 2019 [Available from: https://www.nhs.uk /nhsservices/hospitals/guide-to-nhs-waiting-times-in-england/, .
4. England. N. Consultant-led Referral-to-treatment waiting times 2022/2023 2022 [Available from: https://www.england.nhs.uk /statistics/statistical-work-areas/rtt-waiting-times /rtt-data-2022- .
5. Krelle H, Barclay C, Tallack C. Waiting for care–understanding the pandemic's effects on people's health and quality of life. The Health Foundation; 2021.
6. Trust. N. Elective (planned) treatment waiting times. 2023. [Available from: https://www.nuffieldtrust.org.uk/resource/treatment-waiting-times.
7. TIME. NEGIRF. Surgical hubs 2024 [Available from: https://gettingitrightfirsttime.co.uk/hvlc/surgical-hubs/.
8. Barratt H, Hutchings A, Pizzo E, Aspinal F, Jasim S, Gafoor R, et al. Mixed methods evaluation of the Getting it Right First Time programme in elective orthopaedic surgery in England: an analysis from the National Joint Registry and Hospital Episode Statistics. BMJ open. 2022;12(6):e058316.
9. Briggs T, Kay P, Vig S, Magallanes A, Rehman H, Fleming M, et al. Optimising surgical hubs for staff: case studies on training, wellbeing and retention. British Journal of Healthcare Management. 2022;28(12):1-9.
10. (NIHR). NIfHaCR. 22/26 HSDR Evaluating the High Volume Low Complexity (HVLC) surgical hubs model Commissioning Brief. 2022 [Available from: https://www.nihr.ac.uk/documents/2226-hsdr-evaluating-the-high-volume-low-complexity-hvlc-surgical-hubs-model-commissioning-brief/29940.
11. Scantlebury A, Adamson J, Salisbury C, Brant H, Anderson H, Baxter H, et al. Do general practitioners working in or alongside the emergency department improve clinical outcomes or experience? A mixed-methods study. BMJ open. 2022;12(9):e063495.

For peer review only - http://bmjopen.bmj.com/site/about/guidelines.xhtml

12. Fulop NJ, Ramsay A, Hunter RM, McKevitt C, Perry C, Turner SJ, et al. Evaluation of reconfigurations of acute stroke services in different regions of England and lessons for implementation: a mixed-methods study. Health Services Delivery Research. 2019;7(7).
13. Damschroder LJ, Aron DC, Keith RE, Kirsh SR, Alexander JA, Lowery JC. Fostering implementation of health services research findings into practice: a consolidated framework for advancing implementation science. Implementation science. 2009;4(1):1-15.
14. Damschroder LJ, Reardon CM, Opra Widerquist MA, Lowery J. Conceptualizing outcomes for use with the Consolidated Framework for Implementation Research (CFIR): the CFIR Outcomes Addendum. Implementation science. 2022;17(1):7.
15. Kirk MA, Kelley C, Yankey N, Birken SA, Abadie B, Damschroder L. A systematic review of the use of the Consolidated Framework for Implementation Research. Implementation Science. 2015;11(1):1-13.
16. Merlo G, Page K, Zardo P, Graves N. Applying an implementation framework to the use of evidence from economic evaluations in making healthcare decisions. Applied Health Economics and Health Policy. 2019;17:533-43.
17. Craig P, Cooper C, Gunnell D, Haw S, Lawson K, Macintyre S, et al. Using natural experiments to evaluate population health interventions: new Medical Research Council guidance. J Epidemiol Community Health. 2012;66(12):1182-6.
18. Over 50 new surgical hubs set to open across England to help bust the COVID-19 backlogs [press release]. 2022.
19. Callaway B, Sant'Anna PH. Difference-in-differences with multiple time periods. Journal of econometrics. 2021;225(2):200-30.
20. Siciliani L, Sivey P, Street A. Differences in length of stay for hip replacement between public hospitals, specialised treatment centres and private providers: selection or efficiency? Health economics. 2013;22(2):234-42.
21. Dobkin C, Finkelstein A, Kluender R, Notowidigdo MJ. The economic consequences of hospital admissions. American Economic Review. 2018;108(2):308-52.
22. Protopapa K, Simpson J, Smith N, Moonesinghe S. Development and validation of the surgical outcome risk tool (SORT). Journal of British Surgery. 2014;101(13):1774-83.
23. Scantlebury A, Adamson J. Conducting a large, longitudinal, multi-site qualitative study within a mixed methods evaluation of a UK national health policy: reflections from the GPED study. International Journal of Qualitative Methods. 2022;21:16094069221140873.
24. Research. CFfI. CFIR. 2024 [Available from: https://cfirguide.org/.
25. Sheard L, Marsh C. How to analyse longitudinal data from multiple sources in qualitative health research: the pen portrait analytic technique. BMC Medical Research Methodology. 2019;19(1):1-10.
26. Braun V, Clarke V, Hayfield N, Davey L, Jenkinson E. Doing reflexive thematic analysis. Supporting research in counselling and psychotherapy: Qualitative, quantitative, and mixed methods research: Springer; 2023. p. 19-38.
27. Sheard L. Telling a story or reporting the facts? Interpretation and description in the qualitative analysis of applied health research data: a documentary analysis of peer review reports. SSM-Qualitative Research in Health. 2022;2:100166.
28. Castelli A, Chalkley MJ, Gaughan JM, Rodriguez Santana I. Productivity of the English National Health Service: 2017/18 update. 2020.
29. Carter P. Productivity in NHS Hospitals. Independent report for Department of Health and Social Care. 2015. [Available from: https://www.gov.uk/government/publications/productivity-in-nhs-hospitals.
30. Improvement. N. Carter efficiency guidance.; 2018.
31. England. N. A guide to the market forces factor. . 2021.
32. England. N. Capital guidance 2022 to 2025. 2022b.

For peer review only - http://bmjopen.bmj.com/site/about/guidelines.xhtml

Standards for Reporting Implementation Studies: the StaRI checklist for completion

The StaRI standard should be referenced as: Pinnock H, Barwick M, Carpenter C, Eldridge S, Grandes G, Griffiths CJ, Rycroft-Malone J, Meissner P, Murray E, Patel A, Sheikh A, Taylor SJC for the StaRI Group. Standards for Reporting Implementation Studies (StaRI) statement. *BMJ* 2017;356:i6795

The detailed Explanation and Elaboration document, which provides the rationale and exemplar text for all these items is: Pinnock H, Barwick M, Carpenter C, Eldridge S, Grandes G, Griffiths C, Rycroft-Malone J, Meissner P, Murray E, Patel A, Sheikh A, Taylor S, for the StaRI group. Standards for Reporting Implementation Studies (StaRI). Explanation and Elaboration document. *BMJ Open* 2017 2017;7:e013318

Notes: A key concept of the StaRI standards is the dual strands of describing, on the one hand, the implementation strategy and, on the other, the clinical, healthcare, or public health intervention that is being implemented. These strands are represented as two columns in the checklist.

The primary focus of implementation science is the implementation strategy (column 1) and the expectation is that this will always be completed.	The evidence about the impact of the intervention on the targeted population should always be considered (column 2) and either health outcomes reported or robust evidence cited to support a known beneficial effect of the intervention on the health of individuals or populations.

The StaRI standardsrefers to the broad range of study designs employed in implementation science. Authors should refer to other reporting standards for advice on reporting specific methodological features. Conversely, whilst all items are worthy of consideration, not all items will be applicable to, or feasible within every study.

Checklist item		Reported on page #	Implementation Strategy	Reported on page #	Intervention
			"Implementation strategy" refers to how the intervention was implemented		"Intervention" refers to the healthcare or public health intervention that is being implemented.
**Title and abstract**					
Title	**1**	1	Identification as an implementation study, and description of the methodology in the title and/or keywords		
Abstract	**2**	3	Identification as an implementation study, including a description of the implementation strategy to be tested, the evidence-based intervention being implemented, and defining the key implementation and health outcomes.		
**Introduction**					
Introduction	**3**	5-6	Description of the problem, challenge or deficiency in healthcare or public health that the intervention being implemented aims to address.		
Rationale	**4**	5-6	The scientific background and rationale for the implementation strategy (including any underpinning		The scientific background and rationale for the intervention being implemented (including evidence

			theory/framework/model, how it is expected to achieve its effects and any pilot work).		about its effectiveness and how it is expected to achieve its effects).
Aims and objectives	**5**	7	The aims of the study, differentiating between implementation objectives and any intervention objectives.		
**Methods: description**					
Design	**6**	7-8	The design and key features of the evaluation, (cross referencing to any appropriate methodology reporting standards) and any changes to study protocol, with reasons		
Context	**7**	5-6	The context in which the intervention was implemented. (Consider social, economic, policy, healthcare, organisational barriers and facilitators that might influence implementation elsewhere).		
Targeted 'sites'	**8**	10-16	The characteristics of the targeted 'site(s)' (e.g locations/personnel/resources etc.) for implementation and any eligibility criteria.		The population targeted by the intervention and any eligibility criteria.
Description	**9**	5-6, 10,11, 12, 13	A description of the implementation strategy		A description of the intervention
Sub-groups	**10**	N/A	Any sub-groups recruited for additional research tasks, and/or nested studies are described		
**Methods: evaluation**					
Outcomes	**11**	4	Defined pre-specified primary and other outcome(s) of the implementation strategy, and how they were assessed. Document any pre-determined targets		Defined pre-specified primary and other outcome(s) of the intervention (if assessed), and how they were assessed. Document any pre-determined targets
Process evaluation	**12**	N/A	Process evaluation objectives and outcomes related to the mechanism by which the strategy is expected to work		
Economic evaluation	**13**	11-12, 14-17	Methods for resource use, costs, economic outcomes and analysis for the implementation strategy		Methods for resource use, costs, economic outcomes and analysis for the intervention
Sample size	**14**	10-17	Rationale for sample sizes (including sample size calculations, budgetary constraints, practical considerations, data saturation, as appropriate)		
Analysis	**15**	10-17	Methods of analysis (with reasons for that choice)		

For peer review only

Sub-group analyses	**16**	N/A	Any a priori sub-group analyses (e.g. between different sites in a multicentre study, different clinical or demographic populations), and sub-groups recruited to specific nested research tasks

For peer review only

**Results**					
Characteristics	**17**	N/A	Proportion recruited and characteristics of the recipient population for the implementation strategy		Proportion recruited and characteristics (if appropriate) of the recipient population for the intervention
Outcomes	**18**	N/A	Primary and other outcome(s) of the implementation strategy		Primary and other outcome(s) of the Intervention (if assessed)
Process outcomes	**19**	N/A	Process data related to the implementation strategy mapped to the mechanism by which the strategy is expected to work		
Economic evaluation	**20**	N/A	Resource use, costs, economic outcomes and analysis for the implementation strategy		Resource use, costs, economic outcomes and analysis for the intervention
Sub-group analyses	**21**	N/A	Representativeness and outcomes of subgroups including those recruited to specific research tasks		
Fidelity/ adaptation	**22**	N/A	Fidelity to implementation strategy as planned and adaptation to suit context and preferences		Fidelity to delivering the core components of intervention (where measured)
Contextual changes	**23**	N/A	Contextual changes (if any) which may have affected outcomes		
Harms	**24**	N/A	All important harms or unintended effects in each group		
**Discussion**					
Structured discussion	**25**	N/A	Summary of findings, strengths and limitations, comparisons with other studies, conclusions and implications		
Implications	**26**	N/A	Discussion of policy, practice and/or research implications of the implementation strategy (specifically including scalability)		Discussion of policy, practice and/or research implications of the intervention (specifically including sustainability)
**General**					
Statements	**27**	17-19	Include statement(s) on regulatory approvals (including, as appropriate, ethical approval, confidential use of routine data, governance approval), trial/study registration (availability of protocol), funding and conflicts of interest		

For peer review only

For peer review only - http://bmjopen.bmj.com/site/about/guidelines.xhtml

BMJ Open

Mixed Methods EvAluation of the high-volume low-complexity Surgical hUb pRogrammE (MEASURE): a mixed methods study protocol

Journal:	*BMJ Open*
Manuscript ID	bmjopen-2024-086338.R1
Article Type:	Protocol
Date Submitted by the Author:	22-Mar-2024
Complete List of Authors:	Scantlebury, Arabella; University of York, York Trials Unit, Department of Health Sciences<br>Sivey, Peter; University of York, Centre for Health Economics<br>Anteneh, Zecharias; University of York, Centre for Health Economics<br>Ayres, Ben; St George's University Hospitals NHS Foundation Trust<br>Bloor, Karen; University of York, Department of Health Sciences<br>Castelli , Adriana; University of York, Centre for Health Economics<br>Castro-Avila, Ana; University of York, Health Sciences<br>Davies, Firoza; University of York<br>Davies, Simon; Hull York Medical School, Centre for Health and Population Sciences<br>Glerum-Brooks, Karen; University of York, York Trials Unit<br>Gutacker, Nils; University of York, Centre for Health Economics<br>Lampard, Pete; University of York, Department of Health Sciences<br>Rangan, Amar; James Cook University Hospital, Trauma and Orthopaedics; University of York, Department of Health Sciences<br>Saad, Ahmed; James Cook University Hospital, Opthamology; Hull York Medical School<br>Street, A; LSE<br>Wen, Jinglin; University of York, Centre for Health Economics<br>Adamson, Joy; University of York, Health Sciences
**Primary Subject Heading**:	Surgery
Secondary Subject Heading:	Health economics, Health policy, Qualitative research, Health services research
Keywords:	HEALTH ECONOMICS, Health policy < HEALTH SERVICES ADMINISTRATION & MANAGEMENT, Protocols & guidelines < HEALTH SERVICES ADMINISTRATION & MANAGEMENT, ORTHOPAEDIC & TRAUMA SURGERY, QUALITATIVE RESEARCH, SURGERY

SCHOLARONE™
Manuscripts

For peer review only - http://bmjopen.bmj.com/site/about/guidelines.xhtml

Mixed Methods EvAluation of the high-volume low-complexity Surgical hUb pRogrammE (MEASURE): a mixed methods study protocol

Authors: Arabella Scantlebury (0000-0003-3518-2740), Peter Sivey (0000-0002-3703-615X), Zecharias Anteneh (0000-0001-6588-9056), Benjamin Ayres (0000-0002-8738-7724), Karen Bloor (0000-0003-4852-9854), Adriana Castelli (0000-0002-2546-419X), Ana Castro Avila (0000-0003-4475-4325), Firoza Davies, Simon Davies (0000-0003-2786-6237), Karen Glerum-Brooks (0000-0001-7871-6293), Nils Gutacker (0000-0002-2833-0621), Pete Lampard (0000-0003-3963-8602), Amar Rangan (0000-0002-5452-8578), Ahmed Saad (0000-0002-5159-8001), Andrew Street (0000-0002-2540-0364), Jinglin Wen (0000-0002-2575-8497), Joy Adamson (0000-0002-9860-0850).

York Trials Unit, Department of Health Sciences, ARRC Building, University of York, York, YO10 5DD, UK, Assistant Professor, Arabella Scantlebury

Centre for Health Economics, University of York, YO10 5DD, UK, Reader, Peter Sivey

Department of Health Sciences, ARRC Building, University of York, York, YO10 5DD, UK,

Centre for Health Economics, University of York, York, YO10 5DD, UK, Research Fellow, Zecharias Anteneh

St George's University Hospitals NHS Trust, London, SW17 0QT, UK, Consultant Urologist, Ben Ayres

Department of Health Sciences, ARRC Building, University of York, York, YO10 5DD, UK, Professor, Karen Bloor

For peer review only

Centre for Health Economics, University of York, YO10 5DD, UK, Senior Research Fellow, Adriana Castelli

Department of Health Sciences, ARRC Building, University of York, York, YO10 5DD, UK, Research Fellow, Ana Castro-Avila

York Trials Unit, Department of Health Sciences, ARRC Building, University of York, York, YO10 5DD, UK, Lay representative, Firoza Davies,

Centre for Health and Population Sciences, Hull York Medical School, Siwards Way, Heslington, York, YO10 5DD, UK, Reader in Perioperative Medicine, Consultant Anaesthetist, Simon Davies

York Trials Unit, Department of Health Sciences, ARRC Building, University of York, York, YO10 5DD, UK, Patient and Public Involvement and Stakeholder Engagement Manager, Karen Glerum Brooks,

Centre for Health Economics, University of York, YO10 5DD, UK, Professor, Nils Gutacker

Department of Health Sciences, ARRC Building, University of York, York, YO10 5DD, UK, Research Fellow, Pete Lampard

York Trials Unit, Department of Health Sciences, ARRC Building, University of York, York, YO10 5DD, UK, Research Fellow, and South Tees Hospital NHS Foundation Trust, The James Cook University Hospital, Middlesbrough, TS4 3BW, UK. Professor and Consultant Orthopaedic Surgeon Amar Rangan.

South Tees Hospital NHS Foundation Trust, The James Cook University Hospital, Middlesbrough, TS4 3BW, UK, Hull York Medical School, Siwards Way, Heslington, York, YO10 5DD, UK, and Zagazig University, Zagazig, P.O Box s44519, Egypt, Consultant and Senior Clinical Lecturer, Ahmed Saad

Department of Health Policy, London School of Economics, London, WC2A 2AE, Professor, Andrew Street

For peer review only

Centre for Health Economics, University of York, YO10 5DD, UK, Research Fellow, Jinglin Wen

York Trials Unit, Department of Health Sciences, ARRC Building, University of York, York, YO10 5DD, UK, Professor, Joy Adamson

Correspondence to: Arabella Scantlebury, York Trials Unit, Department of Health Sciences, University of York, ARRC Building, University of York, York, YO10 5 DD, UK

Arabella.Scantlebury@york.ac.uk

For peer review only

For peer review only - http://bmjopen.bmj.com/site/about/guidelines.xhtml

ABSTRACT

INTRODUCTION: The waiting list for elective surgery in England recently reached over 7.8 million people and waiting time targets have been missed since 2010. The high-volume low complexity (HVLC) surgical hubs programme aims to tackle the backlog of patients awaiting elective surgery treatment in England. This study will evaluate the impact of HVLC surgical hubs on productivity, patient care and the workforce.

METHODS AND ANALYSIS: This 4-year project consists of 6 inter-linked work packages (WPs) and is informed by the Consolidated Framework for Implementation Research. *WP1*: Mapping current and future HVLC provision in England through document analysis, quantitative datasets (e.g., Hospital Episodes Statistics) and interviews with national service leaders. *WP2*: Exploring the effects of HVLC hubs on key performance outcomes, primarily the volume of low-complexity patients treated, using quasi-experimental methods. *WP3*: Exploring the impact and implementation of HVLC hubs on patients, health professionals and the local NHS through approximately nine longitudinal, multi-method qualitative case studies. *WP4*: Assessing productivity of HVLC surgical hubs using the Centre for Health Economics NHS productivity measure and Lord Carter's operational productivity measure. *WP5*: Conducting a mixed-methods appraisal will assess the influence of HVLC surgical hubs on the workforce using: qualitative data (WP3) and quantitative data (e.g. NHS England's workforce statistics and intelligence from WP2). *WP6*: Analysing the costs and consequences of HVLC surgical hubs will assess their achievements in relation to their resource use to establish value for money. A Patient and Public Involvement Group (PPI) will contribute to study design and materials.

ETHICS AND DISSEMINATION: The study has been approved by the East Midlands – Nottingham Research Ethics Committee 23/EM/0231. Participants will provide informed consent for qualitative

For peer review only - http://bmjopen.bmj.com/site/about/guidelines.xhtml

study components. Dissemination plans include multiple academic and non-academic outputs (e.g. Peer-reviewed journals, conferences, social media) and a continuous, feedback-loop of findings to key stakeholders (e.g. NHS England) to influence policy development.

STUDY REGISTRATION: Research registry: Researchregistry9364 (https://www.researchregistry.com/browse-the-registry#home/registrationdetails/64cb6c795cbef8002a46f115/).

STRENGTHS AND LIMITATIONS OF THIS STUDY

- The largest, independent, longitudinal, mixed-methods study of the High-Volume Low Complexity (HVLC) surgical hub programme in England.
- Aims to identify the impact of HVLC surgical hubs on outcomes, productivity, patient care, and the workforce and the differential impact of different service models of HVLC surgical hubs
- Our flexible study design and application of implementation theory will ensure our findings capture local context and are relevant to current policy.
- Quantitative analysis will be constrained by the quantity and quality of available information about the implementation and management of HVLC surgical hubs.
- Models of HVLC surgical hubs are expected to be locally tailored and the policy landscape is complex and shifting; this may present a challenge for analysis.

INTRODUCTION

Globally, health services are experiencing unprecedented levels of demand and are struggling to recover from the effects of the COVID-19 pandemic.(1-4) During 2020, approximately 34% of elective care was cancelled in the UK with elective hip replacements falling to almost zero between March and April 2020.(1, 5) The pandemic came against a back-drop of worsening performance against England's 18-week target for elective surgery since 2011,(6) and improvement post-pandemic has been variable.

For peer review only - http://bmjopen.bmj.com/site/about/guidelines.xhtml
For peer review only

NHS England has launched an elective recovery plan (2) designed specifically to address the "backlog" of patients awaiting routine surgical treatment and to increase elective throughput of the NHS to 130% of pre-pandemic "business as usual" volume by 2024/2025.(2) The high-volume, low-complexity (HVLC) surgical hubs model is part of NHS England's elective recovery blueprint, and has been specifically designed to reduce waiting lists for elective surgery in England. By ring-fencing staff and facilities in designated locations, the HVLC surgical hub programme aims to improve efficiency and productivity in the elective pathway by focussing efforts exclusively on treating low complexity elective patients more quickly. This contrasts with the usual model of elective care where patients with less complex conditions are treated in acute hospitals where surgeons, anaesthetists, nursing and diagnostic staff have competing demands from more complex cases and patients requiring urgent and emergency care.(7)

As of February 2024, NHS England reported that there were over 90 HVLC surgical hubs nationally. This includes "long-standing" HVLC hubs that have been operational since before the official launch of the programme in 2022 and "new" surgical hubs established subsequently. In 2023, the Royal College of Surgeons in collaboration with the Royal College of Anaesthetists developed a non-mandatory accreditation scheme, which aims to ensure national standards for HVLC surgical hubs, with 31 hubs receiving as of January 2024. Plans for the HVLC programme continue to evolve. It is expected that in addition to further HVLC surgical hubs being set-up and accredited that the type of patients directed to HVLC surgical hubs for treatment will expand. This evolution is designed to ensure that the programme responds adequately to changing local and national demands on elective care.(7)

There has been no independent evaluation of the HVLC surgical hubs policy published in peer-reviewed journals. We are aware of two on-going, small-scale evaluations of the HVLC surgical hub programme: a qualitative study led by THIS Institute and a quantitative study led by the Health Foundation. We are working closely with these teams to ensure our efforts are collaborative and complementary. Barratt and colleagues conducted a mixed-method evaluation of the "Getting it Right

For peer review only

For peer review only - http://bmjopen.bmj.com/site/about/guidelines.xhtml

134 First Time (GIRFT) programme in elective orthopaedic surgery (8) and Briggs and colleagues describe
135 a case report of four HVLC surgical hubs. Both studies report positive impacts across several outcomes,
136 providing the rationale for scaling-up of the programme and for a more comprehensive evaluation.
137 (9) Our study (MEASURE) builds on this emerging, but small, evidence base, by providing a four-year,
138 independent, national evaluation of the HVLC surgical hub programme in England, commissioned by
139 the National Institute for Health and Care Research (NIHR).(10)

140 Research question

141 What is the impact of the HVLC hubs on productivity, patient care, and the workforce and what is the
142 differential impact of different service models of HVLC surgical hubs?

143 A summary of the specific aims and objectives, WPs and methods are provided in table 1.

144 **Table 1.** Research objectives, work packages

**Research objectives**	Work Package(s)
To characterise the implementation timing, scale, scope and staffing of HVLC hubs currently working/being set up in England	1
To determine the impact of HVLC hubs on performance in terms of equity of access and uptake, and indicators including volume of activity, patients' length of stay, waiting times, and productivity across different patient populations	2, 3, 4, 6
To explore the impact of HVLC models on professionals working in the hubs (including training, workload, skill-mix, turnover, absence, satisfaction, well-being and attitudes to and scope of practice)	3, 5
To explore the impact of HVLC hubs on the wider local NHS including spillovers in other areas of the NHS (e.g. emergency care); workforce issues across the wider trust(s)	2, 3, 4, 5
To assess the impact of HVLC hubs on patients and carers (including views on travel/transport, nature/suitability, accessibility of premises, and satisfaction, patient reported outcome measures (PROMs) where available). The impact of hubs on the patient pathway, continuity of pre- and post-surgical care, and outpatient appointments will also be considered	2, 3
To explore the implementation of HVLC hubs – how have changes been enacted and experienced and what are the barriers and facilitators to implementation	1, 3
To compare resource utilisation and costs of care across different service models and typologies of HVLC hubs, conduct a cost-impact analysis and establish value for money	1, 2, 6

For peer review only - http://bmjopen.bmj.com/site/about/guidelines.xhtml

METHODS AND ANALYSIS

Study design

This mixed-methods study consists of six inter-linked work packages (WPs) (Figure 1). Our study design is consistent with other recent large-scale evaluations of national health policies (8, 11, 12) and our prior experience of undertaking national mixed-methods health policy evaluations has taught us that our study design must be flexible and able to adapt to shifting policy landscapes and priorities. Over the lifetime of our project, we anticipate changes in both the number of HVLC hubs that will be established and their service remit. We are mindful that the criteria for which patients are treated within hubs may be relaxed and/or expanded in response to changing influences on NHS demand. For example, some HVLC hubs are now inclusive of paediatrics. The individual work packages that are outlined below have been designed with this flexibility in mind to enable us to be responsive and to reflect evolving policy priorities.

Theoretical framework

MEASURE will be informed by the Consolidated Framework for Implementation Research (CFIR),(13, 14) which has increasingly and successfully been applied to evaluate care model redesigns (15) and to underpin mixed method(15) and economic evaluations.(16) The CFIR provides a comprehensive taxonomy of influencing factors that can be used to explore what works, where and why, across multiple contexts. The application of the CFIR to frame the MEASURE study is crucial for understanding the complexities surrounding the implementation of the HVLC programme and particularly for capturing the influence of local context on the programme's implementation and impact. We will use the updated version of the CFIR, which in addition to the 5 core domains (intervention characteristics, wider context, local context, characteristics of the individuals involved and the process of

For peer review only - http://bmjopen.bmj.com/site/about/guidelines.xhtml

168 implementation) includes innovation outcomes (e.g. the 'success or failure of innovation or HVLC hubs').(14)

170 ## Secondary data sources for quantitative analysis

171 Our quantitative analyses (WPs 1, 2, 4, 5, 6) will use NHS England Hospital Episode Statistics (HES) data and the National Joint Registry (NJR) in conjunction with various other additional data sources. Each data source, its use within the MEASURE project and associated work package is outlined in table 2.

174 All patient data to be analysed are pseudonymised by NHS England or the National Joint Registry (NJR); we will not access any patient-identifiable information. All published outputs will include aggregated statistics, such as those at the level of national averages and organisation (i.e. hospital trust and where possible surgical hub), with subgroup analysis at provider or commissioner levels or geographical areas or patient characteristics. No information will be published about individual patients or clinicians or that permits their identification.

180 **Table 2.** Data sources and how they will be used in the MEASURE study

Data set	Use for MEASURE study	Work Package
NHS England Hospital Episode Statistics (HES) data.	Measuring processes and outcomes including volume of surgeries, waiting times, length of stay, readmission, emergency and acute care performance; risk classification and adjustment using information on demographics, co-morbidities and history of health care use.	2,4,6
National Joint Registry	Additional risk classification and stratification for orthopaedic patients using information on Body Mass Index (BMI), American Society of Anaesthesiologists (ASA) ASA score. Health outcomes including revision surgeries.	2, 4, 6
Civil Registrations of deaths out of hospital (Secondary Cut),	Measuring mortality as an outcome.	2, 4, 6

For peer review only - http://bmjopen.bmj.com/site/about/guidelines.xhtml

For peer review only

Patient Reported Outcome Measures (PROMs)	Pre-operative health and health outcomes for hip and knee replacement patients.	2, 4, 6
National Cost Collection	Measuring unit costs of elective care, and other care delivered by Trusts	4, 6
NHS workforce statistics, and Trust annual accounts consolidation data (TACs)	NHS workforce statistics include monthly numbers of NHS Hospital staff working in Trusts and other organisations. TACs include information on NHS Trusts income and expenditure items, received and incurred by type of inputs (labour, materials and capital). These datasets will be used to calculate, where possible, inputs used by surgical hubs.	4, 5, 6
ONS National Life Tables	Post-treatment life expectancy outcomes, to be used to quality adjust output produced by surgical hubs	4
Model Health System' dataset	Hospital site-level efficiency measures such as theatre usage time.	2, 4, 6

Work Package 1: describe and classify current and planned models of HVLC hubs in England

To provide an accurate description of the HVLC surgical hubs programme three integrated phases of work will be conducted in parallel (table 3). This will include describing funding, workforce, the date of commencement of any service change and current service configuration. GIRFT guidance identifies three distinct model types in England: standalone, integrated, ring-fenced.(7) Intelligence obtained in this work package will be crucial for informing our subsequent quantitative and qualitative analyses. For example, dates of service change are essential for our quasi-experimental analysis (WP2) and model types and their impact on hub activity will be explored in-depth in WP2-4. This “live database”

For peer review only

For peer review only - http://bmjopen.bmj.com/site/about/guidelines.xhtml

191 will be continuously updated throughout the project to ensure our subsequent data collection and
192 analyses are based on evolving hub implementation and reflects any anticipated changes to
193 definitions of complexity and the changes in service remit of HVLC hubs more broadly.

194 **Table 3.** The three phases of planned work for Work Package 1

Phase	Data type	Output
1: Identifying characteristics of HVLC hubs	Desktop review of documentary evidence (e.g. NHS/government publications and NHS board papers).<br><br>Intelligence obtained through direct links to the HVLC team at department of Health and Social Care and NHS England (e.g. verbal communications and internal monitoring reports).	A regularly updated database of HVLC hub activity in England.
2: Data Verification	Analysis of HES, NJR and Model Health System to explore and verify information about HVLC hubs identified in phase 1 and 3.	Verified information about identification of low-complexity patients and location of surgical hub sites in the data.<br>Definitions of high/low complexity patients and how they can be identified in secondary data sets (to inform WP2 and WP4).
3. Qualitative service leader interviews	Qualitative interviews with 10-15 service leaders including: policy commissioners and those responsible for national delivery of HVLC hubs (e.g. NHS England, DHSC and GIRFT representatives).	A rich contextual description of the development of the HVLC programme and its evolution over time.<br>Hypotheses about the potential effects of HVLC hubs (WP2 and WP4) and identify potential case sites (WP3).

195

For peer review only - http://bmjopen.bmj.com/site/about/guidelines.xhtml

For peer review only

Work Package 2: quantitative empirical evaluation of the effects of HVLC surgical hubs

We will perform a quasi-experimental evaluation of the HVLC hubs programme building on the MRC guidance on methods for the evaluation of complex interventions using observational data.(17) In some cases, such as the 20 named new hubs mentioned in the government press release in August 2022(18)we will have some certainty about when hospital trusts established HVLC surgical hubs and when they became operational. We will use information from the preliminary work and data verification in WP1 to clarify when changes to using a surgical hub model have occurred, and how these changes can be captured in our datasets.

We will use the variation in timing of implementation of HVLC hubs across England using a staggered difference-in-difference methodology.(19) By measuring the change in outcomes in areas when the HVLC hub model is adopted, and comparing these to the change in outcomes in (yet) unaffected areas, we will be able to estimate the intended and unintended effects of the HVLC hub model of care.

In instances where we cannot be sure of the timing of the opening surgical hubs or we cannot be sure that any 'control' areas are unaffected by the reform, we will explore other flexible empirical approaches such as cross-sectional analyses (20)(which do not require variation over time), and event studies(21) (which do not require unaffected control groups). We will also test whether different types of hubs, including stand-alone, integrated or ring-fenced hubs, achieve different outcomes.

We will use NHS England data and the NJR from financial years 2010/11 through to the latest data available. The volume of low-complexity patients treated at hubs and at trusts associated with hubs is the primary outcome. Several secondary outcomes will also be examined including waiting times and length of stay (LOS), additional hospital-level productivity measures, health outcomes and further spillover effects on emergency and acute care. We will analyse all outcomes separately for high and low-complexity patients as defined by objective metrics (eg (22)). Throughout our analysis we will explore the consequences of the HVLC hubs programme for health inequalities by gender, age, ethnicity and socio-economic status.

For peer review only

For peer review only - http://bmjopen.bmj.com/site/about/guidelines.xhtml

Work Package 3 : in-depth, qualitative case studies of purposively selected HVLC hubs across England

We will conduct longitudinal, multi-method qualitative case studies at up to 9 purposively-selected HVLC hubs across England. Our qualitative data collection will be conducted longitudinally alongside the three iterations of our quantitative evaluation (WP2 and WP4). Data collection will consist of: non-participant observational data, semi-structured interviews and documentary analysis.

A detailed overview of our planned qualitative work is provided in our separate qualitative study protocol- (under review) which draws upon published methodological work on designing and analysing "big qualitative studies" in the context of national mixed methods policy evaluations.(23) Central to our case-study design is the need for flexibility in our methods of sampling and data collection to enable us to be responsive to the policy landscape whilst balancing the need for breadth (a large and varied number of case-sites) and depth (exploring case-sites in sufficient detail to understand local context and policy adaptation).(23)

Purposive selection of qualitative case-sites

We will use each iteration of quantitative analysis (WP2 and WP4) to generate a list of potential qualitative case study sites. The study team and the Virtual Study Advisory Group (VSAG) (patients, clinical staff and NHS policymakers) will then work together to select case sites. We anticipate researchers spending 5-7 days at case sites over a two-month period. Data collection will include approximately 12-15 hours of observation (e.g., hub team meetings) and 10-15 interviews with staff (e.g. anaesthetists, surgeons, nurses, hub administrators and local GIRFT co-ordinators, non-medical practitioners within extended surgical teams, surgical trainees and training programme directors), service leaders (e.g. regional elective recovery leads) and patients and/or carers (sampled following INCLUDE principles) per site. Data collection will be informed by the CFIR resources(24) and will focus on understanding: the implementation and impact of the HVLC programme, how hub provision fits

For peer review only - http://bmjopen.bmj.com/site/about/guidelines.xhtml

with wider local surgical provision, workforce issues (WP5) and patient experience of referral and treatment at the hub.

Our analysis will be based on the “pen portrait” approach,(25) designed specifically for large, multi-dimensional qualitative data analysis – we will produce narrative descriptions (pen portraits) presented under the CFIR domains of each hub. Pen portrait data will then be analysed using reflexive thematic analysis.(26) This interpretive(27) analysis will allow detailed exploration within and across case-sites and will be tailored to address our research objectives for our main funder report and other additional qualitative outputs as appropriate.

Work Package 4

We will measure the productivity of the HVLC surgery hubs by means of two well-established measures: (i) the NHS productivity measure developed by the Centre for Health Economics (28)and (ii) the operational productivity measure developed by Lord Carter(29) and used by NHS England in their Model Health System.(30) Our productivity measures will use several different data sources as described in Table 2. Our study period will start from the introduction of the HVLC hubs programme in 2021 to the latest year available.

We will analyse productivity at the level of each hub and within each clinical specialty, data permitting, therefore analysing productivity levels both within and between specialties. We propose to measure labour productivity, and if possible, total factor productivity of the hubs. Productivity is calculated by comparing the total amount of healthcare output to input(s) for each HVLC hub. We will differentiate between types of surgical hub (stand-alone/ring-fenced/integrated) to investigate whether there are differences in terms of productivity levels achieved between types of surgical hubs. We will consult with the PPI group early in the project to inform any refinements of productivity measures for hubs.

Further, the research team will investigate the potential impact of the establishment of HVLC surgery hubs on the wider system, flagging up any potential (unintended) effects on the productivity of other

For peer review only

activity delivered by NHS Trusts. In particular, we will compare the measures of productivity within and between specialty, both for NHS Trusts with and without a hub.

We will adjust the output measure that feeds into the surgical hub productivity measure for changes in measures of quality (e.g. survival, waiting time), with input from the PPI group and the VSAG to reach an agreement on proposed process and health outcome quality indicators (e.g., survival, emergency readmissions, PROMs) to use in our analysis.

The operational productivity measure (Carter)(29) will provide an alternative measure to cross-reference against the findings of the CHE measure of productivity.

Finally, we will investigate the potential drivers of variations between surgical hubs' productivity measures, if any are detected, following some of our previous work.(28, 29) The selection of determinants will be based on past literature, and the GIRFT 'gateway frameworks' metrics included in the Model Health System. Further determinants will be selected based on the specific set-ups of the surgical hubs, as informed by findings from WPs 1, 3, and 5, and as set out in the five domains of the CFIR. The five domains of the CFIR will also be used to help explain findings of the analyses.

Work Package 5

The staffing of HVLC hubs, and the implications of changes in staffing for care delivered in wider Trusts, are crucial contributors to the success of this policy. We will therefore seek to map the teams in place within Trusts with longstanding and newly implemented HVLC hubs; identify wherever possible the staff working in the hubs and any changes in wider service staffing resulting from the introduction of newer hubs; and explore associations between different staffing models and the effectiveness of the HVLC hubs (as measured in WP2 and WP4).

Initially, we will explore existing data sources, including NHS Digital's workforce statistics and data reflecting local labour markets, such as the wages component of the NHS market forces factor.(31) We will request access to the NHS Electronic Staff Record (ESR) which would provide additional

For peer review only - http://bmjopen.bmj.com/site/about/guidelines.xhtml

information but is not routinely accessible for research. We will examine workforce drivers of successful HVLC models by comparing hubs of different scale, team composition and staffing models based on outcome measures derived from WP2 and 4, taking account of the hierarchical nature of the data. We will monitor indicators of workforce wellbeing and sustainability such as rates of recruitment, retention, turnover, vacancies and use of bank and agency staff. Routinely collected staffing data may, however, be limited in detailing the exact size, composition and working practices of teams working across the hubs and wider NHS organisations. Therefore, we will build on WP1 to describe staffing models in place in the hubs, creating a taxonomy of these if there is variation in approaches between Trusts and over time.

This will be coordinated with the primary qualitative data collection in WP3 where we will investigate staffing models in detail, taking into account the local context and the advice of our PPI panel and VSAG. Data collection from the case sites, summarised descriptively in the pen portraits will include workforce information, which will be explored in more depth. We will, for example, examine rotas, how teams in the HLVC hubs work, whether staff work across hubs and other NHS or private sector organisations and where health professionals moved from when the hubs were created. Within the qualitative case-studies, we will also gather data on broader labour market forces and the local contextual factors that impact on workforce for each site, to explore how these external factors have influenced the nature and functioning of the hubs.

As part of the case study analysis, we will explore the impact of surgical hubs on staff training and development, for example, the number of surgical trainees in each hub, the extent to which trainees are embedded in the hub's activity, perceptions of the quality of surgical training delivered within the hubs, training programmes for non-medical practitioners in the extended surgical team and any perceived unintended consequences in relation to training programmes. We will use CFIR to integrate and interpret the quantitative and qualitative data in a mixed-method analysis.

For peer review only - http://bmjopen.bmj.com/site/about/guidelines.xhtml

Work Package 6

We will conduct a cost-consequence analysis of the different HVLC models based on their estimated effects alongside estimated resource use, notably staff, equipment and capital investment. The estimated effects of HVLC models will follow from the data analysis on patient volumes, health outcomes and spillover effects estimated in WP2. Other consequences (e.g. any impact on training practices) will be identified but may not be possible to measure or value meaningfully. Resource use will be derived from the documentation relating to staffing (workforce minimum datasets, and ESR) and capital investment applications,(32) and by collating information across all WPs 1-5, alongside PSSRU unit cost estimates. We anticipate taking a 'non-inferiority' approach to analysis of health outcomes, but if the quantitative analyses in WP2 identify a significant impact of the different HVLC models on patient health outcomes (e.g. mortality, readmissions, PROMS), we will explore the feasibility of further health economic modelling.

Mixed methods analysis to integrate qualitative and quantitative data across all work packages

Our separate quantitative and qualitative analyses will be jointly displayed in a mixed-methods matrix according to the relevant domains of the CFIR. This will enable a higher-level synthesis of our findings, that draws meta-inferences from both sources of data that are tailored to our overall study objectives and underpinned by relevant theory. This process of using implementation models to integrate large, complex quantitative and qualitative data has been successfully adopted in other large-scale policy evaluations.(11, 12)

Patient and public involvement

Given the complexity of our project and its duration, our PPI contributors are crucial for providing meaningful and active contributions to our study design and key decision-making. For instance, they

For peer review only - http://bmjopen.bmj.com/site/about/guidelines.xhtml

will assist with the selection of quantitative outcomes and qualitative case study sites. We will speak with PPI contributors from a range of backgrounds, locations, genders, ages. They will include both carers and patients who are either on the waiting list for, or have undergone treatments that hubs are focusing on. We will also speak with PPI contributors based around our qualitative case study sites, who will help us to understand local context and services which may be affected by HVLC hubs to tailor our study materials, data collection, analysis and dissemination plans to their area.

ETHICS AND DISSEMINATION

The study is registered through the research registry and qualitative components have been approved by East Midlands-Nottingham Research Ethics Committee 23/EM/0231. University of York are the study sponsor. Participants will provide informed consent for qualitative study components (WP 1, 3, 6). Routine care is not altered by the study and it does not raise significant ethical issues. Quantitative data analysis will involve secondary analysis of pseudonymised datasets available through a programme-level agreement between the Centre for Health Economics (University of York) and NHS England, and a separate data application to the National Joint Registry. Further datasets to be used in the quantitative analysis to inform resource use, labour, capital, and intermediate inputs, will be the Electronic Staff Records (ESR), including pay-roll data, and the Trust Account Consolidation (TACs), i.e. Trusts financial accounts. Access to the Model Health System' dataset, NHS England, will also be sought, as it contains more granular data on theatre productivity, and resource use, including the staffing and skill-mix of surgical hubs.

The study commenced in May 2023 and is expected to run for 48 months.

Dissemination will include academic outputs (e.g., conference presentations, peer-reviewed articles) and lay outputs (e.g., policy briefs, project website, press releases and blog posts) disseminated through social media and our VSAG. We are in regular contact with HVLC programme leads at NHS England, through which (along with contacts at DHSC, Royal College of Surgeons and GIRFT) we plan

For peer review only - http://bmjopen.bmj.com/site/about/guidelines.xhtml

to foster a process of live-feedback throughout the project to ensure our findings and study design is considerate of and influences policy development. This is our most direct route to impact.

For peer review only

For peer review only - http://bmjopen.bmj.com/site/about/guidelines.xhtml

ACKNOWLEDGEMENTS

We would like to thank our public contributors, Ms Elena Bechberger and Professor Tim Briggs for their support and collaboration with the MEASURE study.

CONTRIBUTORS

JA and PS are the study's Co-Chief Investigators. JA, PS, AC, AS, KB are work package leads. The research question was developed and the study was designed by JA, KB, AC, SD, KG-B, NG, AR, PS, ASc, and ASt. ASc drafted the manuscript. The protocol was developed and refined by JA, BA, ZA, KB, AC, AC-A, FD, SD, KG-B, NG, PL, AR, PS, ASc, ASt, and JW. All authors have reviewed, revised and approved the final manuscript.

FUNDING

This work was supported by the National Institute for Health Research, Health Services and Delivery Research Programme (NIHR HS&DR), grant number 153387. The views expressed are those of the authors and not necessarily those of the NIHR or the Department of Health and Social Care.

COMPETING INTERESTS

BA is the Clinical Director of Urology for NHS London and has been involved in the initial implementation of the HVLC surgical hubs in London. The authors declare no other competing interests.

REFERENCES

1. Burn S, Propper, C., Stoye, G., Warner, M., Aylin, P., Bottle., A. What happened to English NHS Hospital activity during the COVID-19 pandemic? : IFS Briefing Note. BN328; 2021 [
2. England N. Delivery plan for tacking the covid-19 backlog of elective care. . 2022a.
3. England. N. Guide to NHS Waiting times in England. 2019 [Available from: https://www.nhs.uk /nhsservices/hospitals/guide-to-nhs-waiting-times-in-england/, .

For peer review only - http://bmjopen.bmj.com/site/about/guidelines.xhtml

4. England. N. Consultant-led Referral-to-treatment waiting times 2022/2023 2022 [Available from: https://www.england.nhs.uk /statistics/statistical-work-areas/rtt-waiting-times /rtt-data-2022-.
5. Krelle H, Barclay C, Tallack C. Waiting for care–understanding the pandemic's effects on people's health and quality of life. The Health Foundation; 2021.
6. Trust. N. Elective (planned) treatment waiting times. 2023. [Available from: https://www.nuffieldtrust.org.uk/resource/treatment-waiting-times.
7. TIME. NEGIRF. Surgical hubs 2024 [Available from: https://gettingitrightfirsttime.co.uk/hvlc/surgical-hubs/.
8. Barratt H, Hutchings A, Pizzo E, Aspinal F, Jasim S, Gafoor R, et al. Mixed methods evaluation of the Getting it Right First Time programme in elective orthopaedic surgery in England: an analysis from the National Joint Registry and Hospital Episode Statistics. BMJ open. 2022;12(6):e058316.
9. Briggs T, Kay P, Vig S, Magallanes A, Rehman H, Fleming M, et al. Optimising surgical hubs for staff: case studies on training, wellbeing and retention. British Journal of Healthcare Management. 2022;28(12):1-9.
10. (NIHR). NIfHaCR. 22/26 HSDR Evaluating the High Volume Low Complexity (HVLC) surgical hubs model Commissioning Brief. 2022 [Available from: https://www.nihr.ac.uk/documents/2226-hsdr-evaluating-the-high-volume-low-complexity-hvlc-surgical-hubs-model-commissioning-brief/29940.
11. Scantlebury A, Adamson J, Salisbury C, Brant H, Anderson H, Baxter H, et al. Do general practitioners working in or alongside the emergency department improve clinical outcomes or experience? A mixed-methods study. BMJ open. 2022;12(9):e063495.
12. Fulop NJ, Ramsay A, Hunter RM, McKevitt C, Perry C, Turner SJ, et al. Evaluation of reconfigurations of acute stroke services in different regions of England and lessons for implementation: a mixed-methods study. Health Services Delivery Research. 2019;7(7).
13. Damschroder LJ, Aron DC, Keith RE, Kirsh SR, Alexander JA, Lowery JC. Fostering implementation of health services research findings into practice: a consolidated framework for advancing implementation science. Implementation science. 2009;4(1):1-15.
14. Damschroder LJ, Reardon CM, Opra Widerquist MA, Lowery J. Conceptualizing outcomes for use with the Consolidated Framework for Implementation Research (CFIR): the CFIR Outcomes Addendum. Implementation science. 2022;17(1):7.
15. Kirk MA, Kelley C, Yankey N, Birken SA, Abadie B, Damschroder L. A systematic review of the use of the Consolidated Framework for Implementation Research. Implementation Science. 2015;11(1):1-13.
16. Merlo G, Page K, Zardo P, Graves N. Applying an implementation framework to the use of evidence from economic evaluations in making healthcare decisions. Applied Health Economics and Health Policy. 2019;17:533-43.
17. Craig P, Cooper C, Gunnell D, Haw S, Lawson K, Macintyre S, et al. Using natural experiments to evaluate population health interventions: new Medical Research Council guidance. J Epidemiol Community Health. 2012;66(12):1182-6.
18. Over 50 new surgical hubs set to open across England to help bust the COVID-19 backlogs [press release]. 2022.
19. Callaway B, Sant'Anna PH. Difference-in-differences with multiple time periods. Journal of econometrics. 2021;225(2):200-30.
20. Siciliani L, Sivey P, Street A. Differences in length of stay for hip replacement between public hospitals, specialised treatment centres and private providers: selection or efficiency? Health economics. 2013;22(2):234-42.
21. Dobkin C, Finkelstein A, Kluender R, Notowidigdo MJ. The economic consequences of hospital admissions. American Economic Review. 2018;108(2):308-52.
22. Protopapa K, Simpson J, Smith N, Moonesinghe S. Development and validation of the surgical outcome risk tool (SORT). Journal of British Surgery. 2014;101(13):1774-83.

For peer review only - http://bmjopen.bmj.com/site/about/guidelines.xhtml

23. Scantlebury A, Adamson J. Conducting a large, longitudinal, multi-site qualitative study within a mixed methods evaluation of a UK national health policy: reflections from the GPED study. International Journal of Qualitative Methods. 2022;21:16094069221140873.
24. Research. CFfI. CFIR. 2024 [Available from: https://cfirguide.org/.
25. Sheard L, Marsh C. How to analyse longitudinal data from multiple sources in qualitative health research: the pen portrait analytic technique. BMC Medical Research Methodology. 2019;19(1):1-10.
26. Braun V, Clarke V, Hayfield N, Davey L, Jenkinson E. Doing reflexive thematic analysis. Supporting research in counselling and psychotherapy: Qualitative, quantitative, and mixed methods research: Springer; 2023. p. 19-38.
27. Sheard L. Telling a story or reporting the facts? Interpretation and description in the qualitative analysis of applied health research data: a documentary analysis of peer review reports. SSM-Qualitative Research in Health. 2022;2:100166.
28. Castelli A, Chalkley MJ, Gaughan JM, Rodriguez Santana I. Productivity of the English National Health Service: 2017/18 update. 2020.
29. Carter P. Productivity in NHS Hospitals. Independent report for Department of Health and Social Care. 2015. [Available from: https://www.gov.uk/government/publications/productivity-in-nhs-hospitals.
30. Improvement. N. Carter efficiency guidance.; 2018.
31. England. N. A guide to the market forces factor. . 2021.
32. England. N. Capital guidance 2022 to 2025. 2022b.

For peer review only - http://bmjopen.bmj.com/site/about/guidelines.xhtml

FIGURE TITLE

Figure 1. Study overview: the six integrated Work Packages of the MEASURE study

For peer review only

For peer review only - http://bmjopen.bmj.com/site/about/guidelines.xhtml

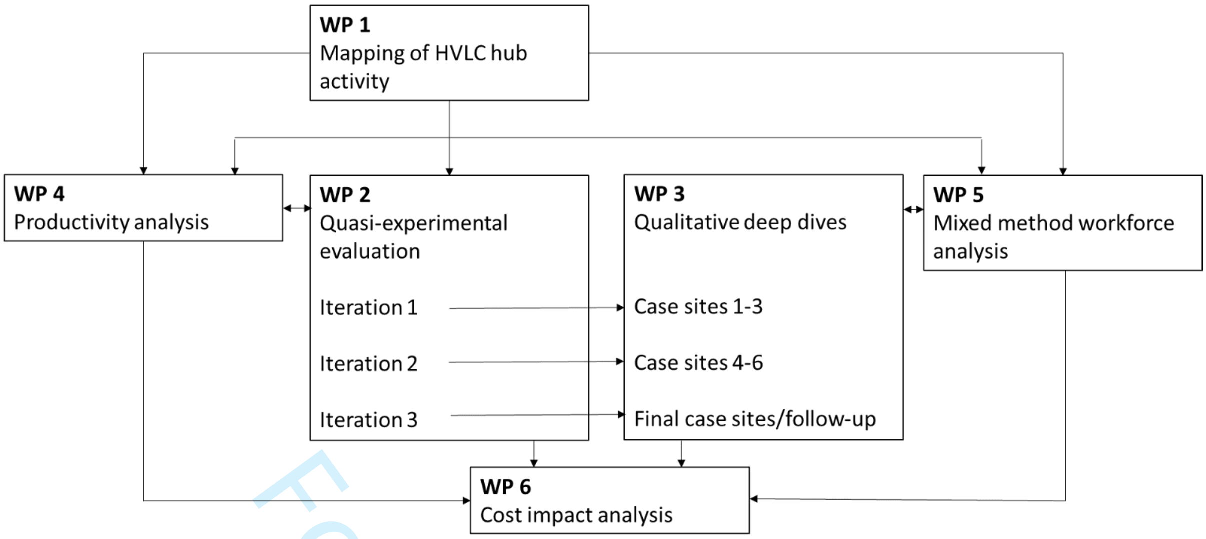

Figure 1 The Six integrated Work Packages of the MEASURE study

For peer review only

Standards for Reporting Implementation Studies: the StaRI checklist for completion

The StaRI standard should be referenced as: Pinnock H, Barwick M, Carpenter C, Eldridge S, Grandes G, Griffiths CJ, Rycroft-Malone J, Meissner P, Murray E, Patel A, Sheikh A, Taylor SJC for the StaRI Group. Standards for Reporting Implementation Studies (StaRI) statement. *BMJ* 2017;356:i6795

The detailed Explanation and Elaboration document, which provides the rationale and exemplar text for all these items is: Pinnock H, Barwick M, Carpenter C, Eldridge S, Grandes G, Griffiths C, Rycroft-Malone J, Meissner P, Murray E, Patel A, Sheikh A, Taylor S, for the StaRI group. Standards for Reporting Implementation Studies (StaRI). Explanation and Elaboration document. *BMJ Open* 2017 2017;7:e013318

Notes: A key concept of the StaRI standards is the dual strands of describing, on the one hand, the implementation strategy and, on the other, the clinical, healthcare, or public health intervention that is being implemented. These strands are represented as two columns in the checklist.

The primary focus of implementation science is the implementation strategy (column 1) and the expectation is that this will always be completed.	The evidence about the impact of the intervention on the targeted population should always be considered (column 2) and either health outcomes reported or robust evidence cited to support a known beneficial effect of the intervention on the health of individuals or populations.

The StaRI standardsrefers to the broad range of study designs employed in implementation science. Authors should refer to other reporting standards for advice on reporting specific methodological features. Conversely, whilst all items are worthy of consideration, not all items will be applicable to, or feasible within every study.

Checklist item		Reported on page #	Implementation Strategy	Reported on page #	Intervention
			"Implementation strategy" refers to how the intervention was implemented		"Intervention" refers to the healthcare or public health intervention that is being implemented.
**Title and abstract**					
Title	**1**	1	Identification as an implementation study, and description of the methodology in the title and/or keywords		
Abstract	**2**	3	Identification as an implementation study, including a description of the implementation strategy to be tested, the evidence-based intervention being implemented, and defining the key implementation and health outcomes.		
**Introduction**					
Introduction	**3**	5-6	Description of the problem, challenge or deficiency in healthcare or public health that the intervention being implemented aims to address.		
Rationale	**4**	5-6	The scientific background and rationale for the implementation strategy (including any underpinning		The scientific background and rationale for the intervention being implemented (including evidence

For peer review only - http://bmjopen.bmj.com/site/about/guidelines.xhtml

			theory/framework/model, how it is expected to achieve its effects and any pilot work).		about its effectiveness and how it is expected to achieve its effects).
Aims and objectives	**5**	7	The aims of the study, differentiating between implementation objectives and any intervention objectives.		
**Methods: description**					
Design	**6**	7-8	The design and key features of the evaluation, (cross referencing to any appropriate methodology reporting standards) and any changes to study protocol, with reasons		
Context	**7**	5-6	The context in which the intervention was implemented. (Consider social, economic, policy, healthcare, organisational barriers and facilitators that might influence implementation elsewhere).		
Targeted 'sites'	**8**	10-16	The characteristics of the targeted 'site(s)' (e.g locations/personnel/resources etc.) for implementation and any eligibility criteria.		The population targeted by the intervention and any eligibility criteria.
Description	**9**	5-6, 10,11, 12, 13	A description of the implementation strategy		A description of the intervention
Sub-groups	**10**	N/A	Any sub-groups recruited for additional research tasks, and/or nested studies are described		
**Methods: evaluation**					
Outcomes	**11**	4	Defined pre-specified primary and other outcome(s) of the implementation strategy, and how they were assessed. Document any pre-determined targets		Defined pre-specified primary and other outcome(s) of the intervention (if assessed), and how they were assessed. Document any pre-determined targets
Process evaluation	**12**	N/A	Process evaluation objectives and outcomes related to the mechanism by which the strategy is expected to work		
Economic evaluation	**13**	11-12, 14-17	Methods for resource use, costs, economic outcomes and analysis for the implementation strategy		Methods for resource use, costs, economic outcomes and analysis for the intervention
Sample size	**14**	10-17	Rationale for sample sizes (including sample size calculations, budgetary constraints, practical considerations, data saturation, as appropriate)		
Analysis	**15**	10-17	Methods of analysis (with reasons for that choice)		

Sub-group analyses	**16**	N/A	Any a priori sub-group analyses (e.g. between different sites in a multicentre study, different clinical or demographic populations), and sub-groups recruited to specific nested research tasks

For peer review only

**Results**					
Characteristics	**17**	N/A	Proportion recruited and characteristics of the recipient population for the implementation strategy		Proportion recruited and characteristics (if appropriate) of the recipient population for the intervention
Outcomes	**18**	N/A	Primary and other outcome(s) of the implementation strategy		Primary and other outcome(s) of the Intervention (if assessed)
Process outcomes	**19**	N/A	Process data related to the implementation strategy mapped to the mechanism by which the strategy is expected to work		
Economic evaluation	**20**	N/A	Resource use, costs, economic outcomes and analysis for the implementation strategy		Resource use, costs, economic outcomes and analysis for the intervention
Sub-group analyses	**21**	N/A	Representativeness and outcomes of subgroups including those recruited to specific research tasks		
Fidelity/ adaptation	**22**	N/A	Fidelity to implementation strategy as planned and adaptation to suit context and preferences		Fidelity to delivering the core components of intervention (where measured)
Contextual changes	**23**	N/A	Contextual changes (if any) which may have affected outcomes		
Harms	**24**	N/A	All important harms or unintended effects in each group		
**Discussion**					
Structured discussion	**25**	N/A	Summary of findings, strengths and limitations, comparisons with other studies, conclusions and implications		
Implications	**26**	N/A	Discussion of policy, practice and/or research implications of the implementation strategy (specifically including scalability)		Discussion of policy, practice and/or research implications of the intervention (specifically including sustainability)
**General**					
Statements	**27**	17-19	Include statement(s) on regulatory approvals (including, as appropriate, ethical approval, confidential use of routine data, governance approval), trial/study registration (availability of protocol), funding and conflicts of interest		

For peer review only
