# Supplementary material for: Mixed Methods EvAluation of the high-volume low-complexity Surgical hUb pRogrammE (MEASURE): a mixed methods study protocol
Source: BMJ Open. 2024 Apr 19;14(4):e086338. doi: 10.1136/bmjopen-2024-086338 (PMC11033628; doi:10.1136/bmjopen-2024-086338)
Supplement: Reviewer comments [file bmjopen-2024-086338.reviewer_comments.pdf]

PEER REVIEW HISTORY

BMJ Open publishes all reviews undertaken for accepted manuscripts. Reviewers are asked to complete a checklist review form (**http://bmjopen.bmj.com/site/about/resources/checklist.pdf**) and are provided with free text boxes to elaborate on their assessment. These free text comments are reproduced below.

ARTICLE DETAILS

**TITLE (PROVISIONAL)**	Mixed Methods EvAluation of the high-volume low-complexity Surgical hUb pRogrammE (MEASURE): a mixed methods study protocol
**AUTHORS**	Scantlebury, Arabella; Sivey, Peter; Anteneh, Zecharias; Ayres, Ben; Bloor, Karen; Castelli, Adriana; Castro-Avila, Ana; Davies, Firoza; Davies, Simon; Glerum-Brooks, Karen; Gutacker, Nils; Lampard, Pete; Rangan, Amar; Saad, Ahmed; Street, A; Wen, Jinglin; Adamson, Joy

This article was not externally reviewed at BMJ Open. Protocols that have been independently assessed prior to submission to BMJ Open are usually fast-tracked to publication on the grounds that further substantial changes will not be possible. This independent assessment will usually be external, independent review for both a substantial grant award from a non-commercial or government funder and ethics approval.
